# Better data for decision-making through Bayesian imputation of suppressed provisional COVID-19 death counts

**DOI:** 10.1371/journal.pone.0288961

**Published:** 2023-08-03

**Authors:** Szu-Yu Zoe Kao, M. Shane Tutwiler, Donatus U. Ekwueme, Benedict I. Truman

**Affiliations:** 1 Division of Cancer Prevention and Control, National Center for Chronic Disease Prevention and Health Promotion, Centers for Disease Control and Prevention, Atlanta, Georgia, United States of America; 2 Alan Shawn Feinstein College of Education, University of Rhode Island, Kingston, Rhode Island, United States of America; 3 National Center for HIV, Viral Hepatitis, STD, and TB Prevention, Centers for Disease Control and Prevention, Atlanta, Georgia, United States of America; UCL: University College London, UNITED KINGDOM

## Abstract

**Purpose:**

To facilitate use of timely, granular, and publicly available data on COVID-19 mortality, we provide a method for imputing suppressed COVID-19 death counts in the National Center for Health Statistic’s 2020 provisional mortality data by quarter, county, and age.

**Methods:**

We used a Bayesian approach to impute suppressed COVID-19 death counts by quarter, county, and age in provisional data for 3,138 US counties. Our model accounts for multilevel data structures; numerous zero death counts among persons aged <50 years, rural counties, early quarters in 2020; highly right-skewed distributions; and different levels of data granularity (county, state or locality, and national levels). We compared three models with different prior assumptions of suppressed COVID-19 deaths, including noninformative priors (M1), the same weakly informative priors for all age groups (M2), and weakly informative priors that differ by age (M3) to impute the suppressed death counts. After the imputed suppressed counts were available, we assessed three prior assumptions at the national, state/locality, and county level, respectively. Finally, we compared US counties by two types of COVID-19 death rates, crude (CDR) and age-standardized death rates (ASDR), which can be estimated only through imputing suppressed death counts.

**Results:**

Without imputation, the total COVID-19 death counts estimated from the raw data underestimated the reported national COVID-19 deaths by 18.60%. Using imputed data, we overestimated the national COVID-19 deaths by 3.57% (95% CI: 3.37%-3.80%) in model M1, 2.23% (95% CI: 2.04%-2.43%) in model M2, and 2.96% (95% CI: 2.76%-3.16%) in model M3 compared with the national report. The top 20 counties that were most affected by COVID-19 mortality were different between CDR and ASDR.

**Conclusions:**

Bayesian imputation of suppressed county-level, age-specific COVID-19 deaths in US provisional data can improve county ASDR estimates and aid public health officials in identifying disparities in deaths from COVID-19.

## Introduction

As of July 2022, more than one million deaths were associated with COVID-19 in the United States [1]. Various publicly available datasets have been used to inform federal, state, and local policies designed to slow the spread of COVID-19 and prevent hospitalizations and deaths. To provide timely data to support different levels of decision-making, the Centers for Disease Control and Prevention’s (CDC) National Center for Health Statistics (NCHS) compiled the mortality data submitted from state health departments to produce provisional national, state, and county COVID-19 mortality data for 2020 [2–5]. Because certain causes of death require more time to review and process, final annual mortality data for a given year are typically released nearly one year later [2, 4]. Prior to release of the final data, provisional data allow researchers to conduct analyses to inform public health policies that can reduce COVID-19 mortality at the local level.

Before provisional data were released to the public, public health researchers often conducted analyses regarding COVID-19 associated deaths using publicly available data compiled by media organizations and data brokers (e.g., *New York Times*, USAFacts) [6–8]. Provisional data published by NCHS have two advantages over daily counts of COVID-19 deaths compiled by media organizations and data brokers [9, 10]. First, state and local governments correct errors (e.g., causes and dates of death, demographic information) in official vital records published in NCHS public-use datasets. In contrast, because data brokers and media organizations compile daily COVID-19 death counts from state and local health department websites, their datasets could contain deaths incorrectly attributed to COVID-19. As a result, datasets published by the *New York Times*, USAFacts, and other data brokers might be less accurate than official provisional datasets published by NCHS. Second, while data brokers and media organizations publish total state or county counts, age is a primary risk factor for COVID-19 mortality [2, 11]. Provisional NCHS datasets summarize age-specific COVID-19 death counts at the county, state, and national levels, which permits further and more informative analyses to support public health decision-making. For example, for COVID-19, age-standardized and crude death rates might have different policy implications. However, because of confidentiality statutes and contractual arrangements with states, NCHS is required to suppress regional, state, and county death counts less than 10 in publicly available tables and reports [3–5]. Although legally and ethically justified, such techniques result in loss of valuable information and might affect the validity of analyses used to inform public health decisions [12–16].

Previous studies have proposed algorithm- and statistical model-based approaches to minimize bias and information loss caused by data suppression required in the data use agreements of public-use datasets [12–17]. Using data from the CDC Wide-ranging Online Data for Epidemiologic Research (CDC WONDER), Tiwari et al. [16] developed an algorithm that imputes county-level, age-specific suppressed mortality rates based on county-level population estimates and state-level mortality rates in the corresponding age group. However, the algorithm does not account for heterogeneity associated with other county-level attributes, and might be of limited utility when high proportions of state-level estimates are also suppressed [15]. In comparison, statistical model-based approaches can account for factors that affect data structures and analyze suppressed data as though it were missing [12, 18]. Bayesian methods have been applied to address problems with missing or suppressed data [19, 20]. Prior investigators used the Poisson-gamma and conditional autoregressive models that account for spatial structure among counties to estimate the distribution of county-level, age-specific mortality rates in CDC WONDER data [11, 12]. In addition, Bayesian methods can flexibly integrate information at different levels of granularity to infer the distributions of suppressed and unsuppressed data [13, 21].

The 2020 NCHS county-level COVID-19 death data by quarter and age pose a unique statistical challenge because of the nature of the pandemic [4]. Previous studies addressed data suppression in statistics (e.g., mean, count) that were stable, continuously collected and reported [13–16]. In contrast, COVID-19 death counts increased substantially from quarter 1 to quarter 4 in many US communities during 2020 [1]. Therefore, the dataset contained numerous zero counts in early quarters and an increasing proportion of suppressed data over time. Data suppression in the provisional county-level COVID-19 death counts could hinder decision-making and statistical analyses that measure the impact of COVID-19 mortality among US counties. To account for the underlying distribution of suppressed and unsuppressed COVID-19 death counts by age and county-level attributes, we employed Bayesian methods to impute the suppressed death counts.

In this study, we provide a method for estimating suppressed COVID-19 death counts in the 2020 NCHS county-level provisional US mortality data to support timely analyses of COVID-19 mortality conducted by researchers who do not have access to complete age-specific provisional mortality data, including CDC researchers outside of NCHS and researchers in other government agencies and academia.

## Materials and methods

### Overview

**Fig 1:**
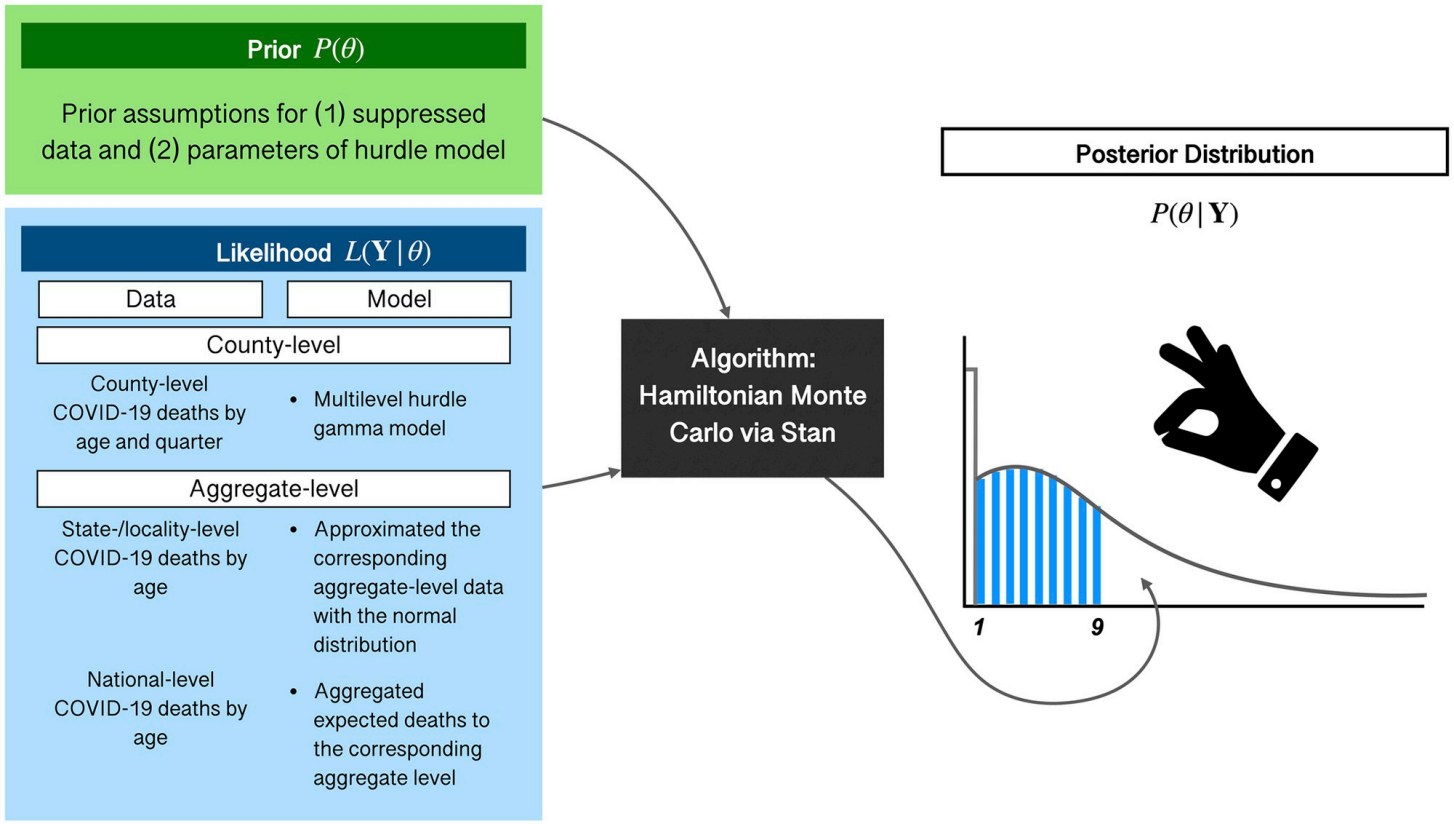
Summary of building the Bayesian multilevel gamma hurdle model.

We developed a Bayesian model to impute suppressed COVID-19 deaths by quarter, county of residence, and age for provisional 2020 US death data. In our Bayesian model, all suppressed data are treated as parameters to be estimated. To improve the precision of our estimates, we used age-specific COVID-19 death counts at different levels of granularity to inform the likelihood, including quarterly data at the county level and annual data at the state and national levels. We evaluated prior knowledge of the plausible values of our estimates of the distribution of suppressed data, including a noninformative prior for all suppressed data (M1), the same weakly informative prior for all ages (M2), and different weakly informative priors for suppressed data by age <50 and ≥50 years (M3). We summarize the relationship between prior, data, likelihood, and posterior distributions in Fig 1. Prior distributions combined with the likelihood (the statistical model summarizing the hypothesized relationship between our measures and COVID-19 deaths) and data result in a posterior distribution that can be evaluated. We compare the three models using performance measures generated at different levels of data aggregation. We demonstrate the importance of age-specific COVID-19 deaths by county by comparing crude (CDR) and age-standardized death rates (ASDR). We used R 4.0.4 and the RStan package to interface between R and Stan, which is a probabilistic programming language for Bayesian inference [22–24]. The model code and datasets are available at https://github.com/syzoekao/COVID19MortImpute.

CDC ethics officials reviewed the study protocol to ensure activities were conducted in compliance with applicable federal law and CDC policy (see e.g., 45 C.F.R. part 46, 21 C.F.R. part 56; 42 U.S.C. §241(d); 5 U.S.C. §552a; 44 U.S.C. §3501 et seq).

## Data

### County-level data

The 2020 provisional data on COVID-19 deaths by quarter, county, and age are available for public use at https://data.cdc.gov/NCHS/AH-Provisional-COVID-19-Deaths-by-Quarter-County-a/ypxr-mz8e [4]. These provisional data are an ad-hoc file including data received and processed by NCHS through April 22, 2021. Although the dataset was later updated in July 2021, we used the dataset released in April 2021 in our analysis. Due to reporting lag, the provisional data released in April 2021 might underestimate the true number of COVID-19 deaths in 2020 [2, 4].

In this provisional dataset, COVID-19 deaths were organized by the county of residence of decedents who died with COVID-19 confirmed or presumed as the underlying or contributing cause of death (ICD-10 code U07.01) [2]. The definition of county—county of residence—used in this provisional dataset differs from that of other NCHS datasets, in which county may be the county of occurrence [25]. Using the decedents’ information (county of residence and age at death), COVID-19 death counts were tabulated by eight age groups (0–17, 18–29, 30–39, 40–49, 50–64, 65–74, 75–84, ≥85) and four quarters for 3,140 US counties in 50 states and the District of Columbia (DC). In this dataset, each row includes a county’s COVID-19 death count for an age group and quarter along with the Federal Information Processing Standard (FIPS) county code, the state, and urban-rural code (S1 Table) [26]. Because of the small number of COVID-19 deaths at ages 0–17 years (199 deaths) in 2020 [2, 11], we excluded this age group from the analysis. For persons aged ≥18 years, the dataset contains 87,920 rows for the COVID-19 death counts by age, quarter, and county (7 age groups x 4 quarters x 3,140 counties).

In this dataset, data elements are suppressed (i.e., not available for analysis) if counts range from one to nine [4]. Only 46 counties have all data elements present for all age groups and quarters because those counties had zero COVID-19 deaths reported in 2020. Overall, 26.6% of data elements were suppressed, ranging from 6.2% in quarter 1 to 41.1% in quarter 4 (S1 Fig). This nearly 7-fold difference between quarters 1 and 4 was because of the increase in the number of counties that had at least one COVID-19 death in 2020.

To impute suppressed COVID-19 death counts for age groups ≥18 years, we considered factors associated with the pattern of both suppressed and unsuppressed COVID-19 deaths, including time (quarter), age group, and urban-rural code (S2 Fig). In addition, we considered age-specific population size at the county level to capture the high correlation between population size and COVID-19 deaths [27]. Age-specific population estimates by county were extracted from the 2015–2019 American Community Survey (ACS) 5-year estimates [28]. Two counties, Wade Hampton Census Area in Alaska (FIPS code: 2270) and Shannon County in South Dakota (FIPS code: 46113), had no matched population estimates from the 2015–2019 ACS, and therefore were removed from the imputation process. The final dataset included 3,138 US counties.

#### Aggregate-level data

We used the NCHS aggregate-level COVID-19 death information to inform the likelihood at the aggregate level (e.g., state- or locality-level, national level) in the Bayesian imputation model (Fig 1) (https://data.cdc.gov/NCHS/Provisional-COVID-19-Deaths-by-Sex-and-Age/9bhg-hcku) [3]. This dataset contains COVID-19 deaths by year, month, sex, and age at the national and state/locality levels, which include all 50 states, New York City, and DC. We used the age-specific COVID-19 death counts by year for both sexes at the state/locality and national levels in this dataset. All age-specific annual COVID-19 deaths were present at the national level, whereas some were suppressed at the state/locality level. To simplify the imputation task in this study and to enable others to reproduce our results, we developed a simple imputation method to impute these suppressed annual COVID-19 deaths at the state/locality level. The details of the simple imputation method for the state-/locality-level data are described in the supplemental methods (S1 File, Supplemental methods 1).

### Developing the Bayesian Model

#### County-level model

We modeled the distribution of COVID-19 deaths by age, quarter, and county using a Bayesian multilevel hurdle gamma model to infer the posterior distributions of the death counts. We made this modeling decision because of the highly right-skewed unsuppressed data with a large mass at zero deaths (gray bars in S3 Fig), and the nested data structure (age groups nested within quarters, quarters nested within counties). The hurdle gamma model estimates the probability of a county having zero or positive deaths using logistic regression [29] and uses the gamma distribution to approximate the highly right-skewed death data among counties reporting ≥1 death count [30, 31]. We did not consider a Poisson distribution or negative binomial distribution to model count data because Stan has constraints on complex modeling for discrete parameters [32]. A multilevel model can account for the relationships between county-level factors and quarterly trends and the correlated data from counties within the same state [33, 34]. The hurdle gamma model was specified as follows:

PYaijt=0|Χ~aijt=1-paijt (1)

PYaijt>0|Χaijt=paijt⋅Gammashape,rateaijt (2)

shape∼Gamma(0.01,0.01) (3)

*Y*_*aijt*_ denotes the COVID-19 death count for age group *a* in county *i* located in state/locality *j* in quarter *t*; *p*_*aijt*_ is defined as the probability that *Y*_*aijt*_ is positive; ${\tilde{Χ}}_{aijt}$ and Χ_*aijt*_ represent the design matrices for the parts of zero and positive death counts, respectively. Based on the gamma distribution, the mean (*μ*) is equal to $\frac{shape}{rate_{aijt}}$. The prior distribution of *shape* was presented in Eq (3). We used a multilevel logistic regression to model *p*_*aijt*_, and considered a log-link function to estimate the mean (*μ*) of the gamma distribution. The detailed specification of the logistic regression for *p*_*aijt*_ and the log-link function for μ are provided in the supplemental methods (S1 File, Supplemental methods 2).

We assumed that suppressed data elements (*y*^*miss*^) followed the gamma distribution in the hurdle model setting. Because of computational constraints, we did not model mechanisms for suppressed data elements separately from unsuppressed ones, but we modeled these data with varying prior assumptions. We considered and compared three model priors, including noninformative priors characterized by a uniform distribution with support ranging from 0.6 to 9.4 (M1); weakly informative priors characterized by truncated normal distributions with mean 1, standard deviation 10, and the same range of support (M2); and weakly informative priors characterized by truncated normal distributions with mean 1, standard deviation differing by age group (5 for ages 18–49 years and 20 for ages ≥50 years), and the same range of support (M3). The range of support, 0.6–9.4, was used because posterior samples were rounded to the nearest integer after the samples were generated from the Bayesian model. Rounding the posterior samples allowed the samples to match the death counts within the suppressed data range, which are non-negative integers ranging from one to nine.


ymiss∼Uniform0.6,9.4 (M1)

ymiss∼Normal1,10,ymiss∈(0.6,9.4) (M2)

yagemiss∼Normal1,5, age<50yearsNormal1,20, age≥50years, yagemiss∈0.6,9.4 (M3)

For M2 and M3, weakly informative priors were centered at 1 because we hypothesized the distribution of the suppressed deaths followed a declining right-skewed distribution [31]. Unlike the other two models, M3 accounted for heterogeneity of COVID-19 deaths attributable to age [2, 11].

#### Integrating aggregate-level information

Using only county-level data might lead to wide and unstable variation in the posterior distribution of suppressed data elements. To reduce the variation, we added aggregate-level information—age-specific annual COVID-19 death counts at the national level and state or locality level—to the likelihood [19]. These aggregate-level data, assumed to be independent, were approximated using normal distributions in Eqs (4) and (5).

Yaj~NormalY^aj,σaj (4)

Ya~NormalY^a,σa (5)

*Y*_*aj*_ and *Y*_*a*_ represent the total annual death counts for age group *a* at the state/locality *j* and national levels, respectively. ${\hat{Y}}_{aj}$ and ${\hat{Y}}_{a}$ are predicted total annual death counts corresponding to *Y*_*aj*_ and *Y*_*a*_, respectively. The parameters *σ*_*aj*_ and *σ*_*a*_ stand for the standard deviations for the state or locality-level and national-level death counts, respectively. Because these aggregate-level data were population counts rather than samples, we did not estimate the standard deviations using traditional epidemiological methods, which infer population parameters from samples. To allow for some degree of uncertainty in the normal approximation, the standard deviations (*σ*_*aj*_ and *σ*_*a*_) were constructed following a rule-based approach. If the national or state or local COVID-19 death count of an age group was more than 5, *σ*_*aj*_ or *σ*_*a*_ was set to 20% of the death count; if the death count was smaller than 5, *σ*_*aj*_ or *σ*_*a*_ was set to 1.

To estimate ${\hat{Y}}_{aj}$ and ${\hat{Y}}_{a}$, we hypothesized that deaths were certified, verified, and counted in the same way that state- and national-level counts are obtained from county-level counts in the real world. We aggregated expected county-level death counts by age and quarter to produce age-specific annual death counts at the state or locality and national levels, respectively. For age group *a* in county *i* located in state or locality *j* in quarter *t*, the predicted death count (${\hat{Y}}_{aijt}$) was a product of the predicted probability of positive death count (${\hat{p}}_{aijt}$) and the expected death count (${\hat{\mu }}_{aijt}$) if a positive death count was observed (Eq 6). The estimation of ${\hat{p}}_{aijt}$ and ${\hat{\mu }}_{aijt}$ is described in the supplemental methods (S1 File, Supplemental methods 2). The predicted total annual death count for age group *a* in state or locality *j* is the sum of the predicted death counts across quarters and counties for age group *a* in state/locality *j* (Eq 7).


Y^aijt=p^aijt⋅μ^aijt (6)

Y^aj=∑t,iY^aijt (7)

The predicted annual death counts for age group *a* at the national level (${\hat{Y}}_{a}$) was calculated in Eq (8).


Y^a=∑t,i,jY^aijt (8)

### Computer simulation

The posterior distribution of the Bayesian imputation model cannot be easily derived. We used Hamiltonian Monte Carlo (HMC) simulation in Stan, which is a probabilistic programming language for Bayesian inference, to simulate samples from the posterior distribution [22]. The HMC estimation process is described in the supplemental methods (S1 File, Supplemental methods 3). After obtaining the fitted model, we simulated COVID-19 deaths by age group, quarter, and county using the fitted model to perform posterior predictive checking, which checks whether the posterior distribution from the fitted model closely approximates the observed data [19, 35]. To ease the computational burden and preserve the uncertainty informed from the Bayesian model, 1,000 sets of imputed suppressed data elements were sampled from the posterior distribution and rounded to integers. Suppressed data elements in the original dataset were replaced with a set of imputed data elements, resulting in 1,000 imputation datasets that combined both imputed suppressed data elements and unsuppressed data elements from the original dataset. We conducted simulations for each prior assumption model (M1–M3). To ensure model convergence, the simulation consisted of 3 chains and 4,000 iterations containing 1,000 warmups for each model.

### Model performance, comparison, and validation

We assessed model performance at the national, state or locality, and data element levels for each fitted model. At the national level, we estimated the percent bias, which measures the percent relative difference of the estimated death counts to the reported death counts by age group and for overall national death counts [36]. At the state/locality level, we estimated the root mean squared error (RMSE) that measures the difference between the predicted and reported counts at the state/locality level [37, 38]. The model performance measures at the national and state/locality levels were generated using the 1,000 datasets by aggregating death counts by county to the corresponding aggregate level. At the data element level, we used the loo package to estimate expected log predictive density through leave-one-out cross-validation (elpd_loo), which measures how well the model performs on new data points [39, 40]. These model performance measures were calculated for each model and were used to compare the model fit among all three models. Details of how the model performance and comparison were conducted are described in the supplemental methods (S1 File, Supplemental methods 4).

### Estimating crude and age-standardized death rates

To show the importance of age-specific COVID-19 deaths in the provisional data, we estimated different types of death rates at the county level in two scenarios. In scenario 1, we assumed that the provisional data were not available and estimated county-level CDR using cumulative daily COVID-19 death counts by county published by the publicly available data source, USAFacts, on December 31, 2020 [9]. In scenario 2, with the provisional data available, we estimated county-level ASDR [41] using the imputation results and unsuppressed data. We investigated the correlation between county-level CDR or ASDR and county social vulnerability, which is measured by the 2018 CDC/ATSDR Social Vulnerability Index (SVI) for all US counties [42–44]. County SVI is a composite indicator that measure different dimensions of county-level emergency preparedness for disastrous events (e.g., minority status, income level, age composition) [42, 43]. The relation between county SVI and the COVID-19 pandemic has been studied to understand the vulnerability of US communities to the pandemic [7, 45]. We hypothesized that county SVI will be more highly correlated with county-level ASDR than county-level CDR. Furthermore, we listed the top 20 counties that were most affected by COVID-19 mortality in 2020 in descending order of county-level CDR and ASDR, respectively.

## Results

### Model performance and comparison

Model convergence and the posterior predictive results are reported in the supplemental results (S2 File, Supplemental results 1). In 2020, national provisional data included 384,375 provisional deaths with COVID-19 as the underlying or contributing cause. The number of deaths reported by age groups are shown in Table 1 (column 1). Using county-level provisional data without imputation, we underestimated the official total number of COVID-19 deaths among US residents aged ≥18 years (N = 384,180) by 18.55%, and consistently underestimated COVID-19 deaths across all age groups, ranging from 12.60% to 76.96%. Using county-level provisional data with imputation, the estimated total number of COVID-19 deaths was 397,910 in M1, 392,767 in M2, and 395,552 in M3. All models overestimated the total number of COVID-19 deaths in the nation by 3.57% (95% CI: 3.37%, 3.80%) for M1, 2.23% (95% CI: 2.05%, 2.43%) for M2, and 2.96% (95% CI: 2.76%, 3.16%) for M3. When comparing death counts by age group across the three models, M1 underestimated death counts the least for persons aged 18–29 years but overestimated death counts the most for the other age groups. For persons aged 40–49 years, in which M1 overestimated deaths by 8.23% (95% CI: 6.70%, 9.84%), M2 and M3 were able to reduce the percent of overestimation to 6.20% (95% CI: 4.70%, 7.74%) and 1.04% (95% CI: –0.41%, 2.40%), respectively. Comparing death counts between M2 and M3, M3 performed better than M2 for those aged 30–49 years, whereas M2 performed better than M3 for other age groups.

**Table 1:** Comparison of national age-specific COVID-19 deaths in 2020 at the national level between reported national estimates, aggregate estimates from provisional county-level data without imputation, and aggregate estimates from provisional county-level data with imputation results from M1, M2, and M3.

	National Estimates of Provisional COVID-19 Deaths in 2020	Aggregate from Provisional County-Level Data Without Imputation		Aggregate from Imputed County-Level COVID-19 Deaths (M1: Noninformative priors for all age groups)		Aggregate from Imputed County-Level COVID-19 Deaths (M2: Same weakly informative prior for all age groups)		Aggregate from Imputed County-Level COVID-19 Deaths (M3: Different weakly informative prior by age groups 18–49 years and ≥50 years)	
Age group	Number of COVID-19 deaths	Aggregate number of COVID-19 deaths	% underestimated without imputation	Aggregate number of COVID-19 deaths	% overestimated with imputation	Aggregate number of COVID-19 deaths	% overestimated with imputation	Aggregate number of COVID-19 deaths	% overestimated with imputation
				[95% credible intervals; CI]	[95% CI]	[95% CI]	[95% CI]	[95% CI]	[95% CI]
18–29	1,476	347	76.96%	1,455	-1.42%	1,438	-2.55%	1,390	-5.83%
				[1,405, 1,508]	[-4.81%, 2.17%]	[1,387, 1,494]	[-6.03%, 1.22%]	[1,344, 1,438]	[-8.94%, -2.57%]
30–39	4,272	1,701	60.18%	4,542	6.33%	4,473	4.71%	4,259	-0.30%
				[4,439, 4,643]	[3.91%, 8.68%]	[4,372, 4,572]	[2.34%, 7.02%]	[4,174, 4,348]	[-2.29%, 1.78%]
40–49	11,291	5,992	46.93%	12,221	8.23%	11,992	6.20%	11,408	1.04%
				[12,047, 12,398]	[6.70%, 9.80%]	[11,822, 12,165]	[4.70%, 7.74%]	[11,245, 11,562]	[-0.41%, 2.40%]
50–64	56,630	42,538	24.88%	59,416	4.92%	58,312	2.97%	59,149	4.45%
				[59,055, 59,759]	[4.28%, 5.53%]	[58,006, 58,632]	[2.43%, 3.54%]	[58,805, 59,488]	[3.84%, 5.05%]
65–74	82,059	65,905	19.69%	85,137	3.75%	83,863	2.20%	84,812	3.35%
				[84,772, 85,478]	[3.31%, 4.17%]	[83,510, 84,183]	[1.77%, 2.59%]	[84,446, 85,197]	[2.91%, 3.82%]
75–84	105,964	89,367	15.66%	109,071	2.93%	107,807	1.74%	108,768	2.65%
				[108,712, 109,430]	[2.59%, 3.27%]	[107,475, 108,173]	[1.43%, 2.08%]	[108,433, 109,110]	[2.33%, 2.97%]
≥85	122,488	107,056	12.60%	126,068	2.92%	124,882	1.95%	125,766	2.68%
				[125,737, 126,407]	[2.65%, 3.20%]	[124,546, 125,217]	[1.68%, 2.23%]	[125,413, 126,110]	[2.39%, 2.96%]
Total	384,180	312,906	18.55%	397,910	3.57%	392,767	2.23%	395,552	2.96%
				[397,121, 398,772]	[3.37%, 3.80%]	[392,039, 393,500]	[2.05%, 2.43%]	[394,790, 396,338]	[2.76%, 3.16%]

At the state or locality level, annual deaths by age group were highly correlated between the reported provisional data and the aggregated data from the imputation results for all three models (ρ = 0.9990 in S4 and S5 Figs). Using RMSE (panel [A] in S2 Table), M2 had the best performance at the state or locality level with the lowest RMSE (mean = 68.03; SD = 1.86). At the county level, we assessed model performance with unsuppressed data elements using Pareto *k* diagnostic values (panel [B] in S2 Table). All models provided estimates consistent with the data for all unsuppressed data elements except for 18 records for M1, 21 records for M2, and 16 records for M3. Most records that the fitted models did not predict well were among older age groups (75–84 years and ≥85 years), quarters 3 and 4, and noncore counties. Based on elpd_loo, M1 performed slightly better than M2 and M3 in providing estimates consistent with unsuppressed data elements. While the three models had slight differences in model performance, the distribution of county-level COVID-19 deaths generated from the posterior distribution were similar across models (S3 Fig).

### Interpretation and application of imputation results

**Fig 2:**
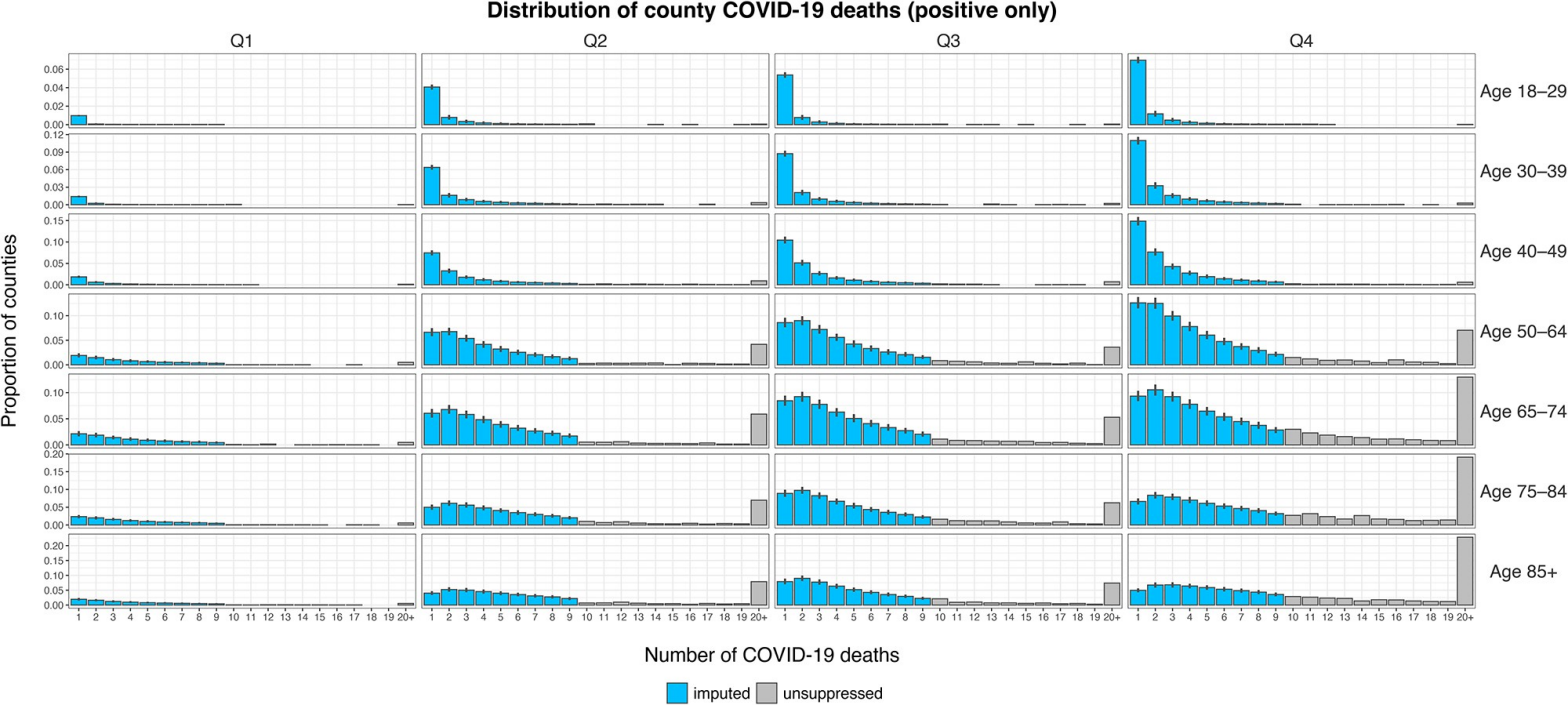
Distribution of county COVID-19 deaths (positive only).

We present the imputation results from M1 as an example to illustrate the interpretation of the results. The proportion of counties by the positive number of COVID-19 deaths (1, 2, …, ≥20 deaths), age group, and quarter calculated from M1 is shown in Fig 2. The distributions incorporating predicted and unsuppressed COVID-19 deaths generally follow a smooth right-skewed shape regardless of quarter or age group. These distributions suggest that among counties reporting positive COVID-19 deaths, the number of COVID-19 deaths that most counties reported was likely to fall within the ranges of suppressed data. Among those aged <65 years, most counties reported one death in all quarters. For those aged ≥65 years, the number of deaths reported by most counties increased from one death in quarter 1 to two or three deaths in quarters 2–4.

The correlation between county SVI and county CDR for COVID-19 in scenario 1 was 0.19, lower than the correlation between county SVI and county ASDR for COVID-19 in scenario 2 (~0.32 for all models) (S6 Fig). In Tables 2 and S3, we presented the top 20 counties most affected by COVID-19 mortality in 2020 by county-level CDR in scenario 1, and ASDR in scenario 2 using the imputation results from M1, M2, and M3. The top 20 counties differed by type of death rate. There were only two counties (Hamlin County, SD and Buffalo County, SD) selected by both CDR and ASDR. Regardless of the type of death rates, most of the top 20 counties were noncore counties or counties with relatively small population size. The top 20 counties selected based on the CDR in scenario 1 had a lower average SVI (40.02) than the average SVI among the top 20 counties selected based on the ASDR in scenario 2 (74.99 for M1 and M3, 77.01 for M2). In addition, we calculated the average county-level CDR and ASDR among all the counties within each US census division to show how geographic distribution of COVID-19 mortality burden might differ between CDR and ASDR (Table 3). All 9 US census divisions were ranked by the mean county-level CDR and ASDR, respectively. Compared between CDR and ASDR, the rankings were different among five divisions. Notably, the ranking of East South Central changed from 4 based on CDR to 2 using ASDR.

**Table 2:** Top 20 counties that were most affected by COVID-19 associated deaths in 2020 using different metrics of COVID-19 death rate: Crude death rate calculated from the death counts reported in USAFacts and age-standardized death rate calculated from the imputation model M1.

USAFacts				M1: Noninformative Uniform Prior for All Age Groups			
County, state (FIPS code)	Urban-rural code	Social Vulnerability Index (SVI)	Crude COVID-19 death rate per 100,000 population	County, state (FIPS code)	Urban-rural code	Social Vulnerability Index (SVI)	Age-standardized COVID-19 death rate per 100,000 population [95% CI]
Gove County, KS (20063)	Noncore	9.75	834.60	Buffalo County, SD (46017)	Noncore	97.10	1,644.21 [1,091.86, 2,521.67]
Jerauld County, SD (46073)	Noncore	5.06	745.16	Armstrong County, TX (48011)	Medium metro	15.99	985.76 [553.79, 1,476.58]
Dickey County, ND (38021)	Noncore	11.59	656.81	Issaquena County, MS (28055)	Noncore	95.10	936.96 [570.23, 1,550.03]
Iron County, WI (55051)	Noncore	36.02	633.02	Martin County, TX (48317)	Small metro	42.39	819.44 [477.30, 1,263.91]
Gregory County, SD (46053)	Noncore	28.47	621.27	Cottle County, TX (48101)	Noncore	93.63	786.89 [414.23, 1,331.21]
Foster County, ND (38031)	Noncore	1.75	591.90	Benson County, ND (38005)	Noncore	84.33	759.76 [526.98, 1,009.55]
Turner County, SD (46125)	Small metro	10.80	584.45	Floyd County, TX (48153)	Noncore	82.99	711.39 [533.93, 918.59]
Emporia city, VA (51595)	Noncore	95.67	561.17	Corson County, SD (46031)	Noncore	83.95	708.93 [411.99, 1,137.43]
Lamb County, TX (48279)	Noncore	89.75	558.44	McKinley County, NM (35031)	Micropolitan	98.89	706.26 [689.14, 725.70]
Hamlin County, SD (46057)	Noncore	15.86	551.59	Cochran County, TX (48079)	Noncore	86.08	705.01 [359.56, 1,182.82]
Galax city, VA (51640)	Noncore	98.38	551.44	Dewey County, SD (46041)	Noncore	88.31	667.96 [377.07, 1,098.33]
Ness County, KS (20135)	Noncore	15.00	545.45	Sherman County, TX (48421)	Noncore	46.11	643.93 [337.21, 1,130.61]
Hancock County, GA (13141)	Micropolitan	79.84	543.93	Maverick County, TX (48323)	Micropolitan	97.42	633.89 [602.75, 664.95]
Pierce County, ND (38069)	Noncore	19.14	528.30	Oldham County, TX (48359)	Medium metro	43.22	623.00 [197.37, 924.17]
Faulk County, SD (46049)	Noncore	7.77	521.97	Hamlin County, SD (46057)	Noncore	15.86	607.63 [536.18, 714.31]
Renville County, ND (38075)	Micropolitan	2.80	515.69	Todd County, SD (46121)	Noncore	95.64	605.50 [363.25, 891.28]
Buffalo County, SD (46017)	Noncore	97.10	509.68	Ziebach County, SD (46137)	Noncore	95.38	602.05 [359.69, 1,023.63]
Grant County, SD (46051)	Noncore	5.51	496.31	Culberson County, TX (48109)	Noncore	99.71	600.87 [326.72, 1,029.50]
Kenedy County, TX (48261)	Micropolitan	73.28	495.05	Dallam County, TX (48111)	Noncore	55.03	598.59 [372.44, 871.42]
Neshoba County, MS (28099)	Noncore	96.85	484.24	Big Horn County, MT (30003)	Noncore	82.74	597.15 [463.38, 762.60]
		**Average SVI among the top 20 counties**				**Average SVI among the top 20 counties**	
		**40.02**				**74.99**	

**Table 3:** Mean and interquartile ranges (IQRs) of county-level crude and age-standardized COVID-19 deaths by US census division.

		USAFacts		Imputation using M1	
US census region	US census division	Mean county-level crude COVID-19 death rate per 100,000 population [IQR]	Ranking by crude COVID-19 death rate	Mean county-level age-standardized COVID-19 death rate per 100,000 population [IQR]	Ranking by age-standardized COVID-19 death rate
Northeast	Division 1: New England	68 [10, 115]	8	73 [20, 130]	8
Northeast	Division 2: Middle Atlantic	114 [60, 147]	5	140 [92, 168]	7
Midwest	Division 3: East North Central	123 [76, 156]	3	153 [117, 184]	4
Midwest	Division 4: West North Central	132 [61, 168]	2	181 [122, 213]	3
South	Division 5: South Atlantic	101 [50, 127]	6	149 [90, 183]	6
South	Division 6: East South Central	123 [66, 164]	4	189 [116, 230]	2
South	Division 7: West South Central	155 [82, 200]	1	246 [161, 300]	1
West	Division 8: Mountain	98 [35, 130]	7	149 [82, 193]	5
West	Division 9: Pacific	40 [16, 57]	9	68 [33, 89]	9

*Note*: Both crude and age-standardized COVID-19 death rates are calculated as the average county-level rates among the counties within the US census division. IQRs represent interquartile ranges, which are the county-level death rates at the 25^th^ and 75^th^ percentiles. US census divisions are ranked by the descending order of the division-specific county-level COVID-19 death rates.

## Discussion

This study illustrates a flexible Bayesian approach to impute suppressed COVID-19 death counts by age, county, and quarter in provisional data. This approach might be useful for facilitating research activities and policy analyses for COVID-19 mortality among public health researchers such as CDC researchers outside of NCHS and researchers in other government agencies and academia. The Bayesian imputation models used different prior assumptions about the unknown distributions of suppressed death counts; integrated provisional death counts at county, state or locality, and national levels; and accounted for excessive zero death counts [13–15, 19–21]. Our study showed that this approach can yield valid and consistent estimates for suppressed data under different prior assumptions. The provisional data combined with the imputed suppressed data elements could be used in further analyses to inform intervention policies to slow the spread of COVID-19.

The Bayesian approach provides the benefit of incorporating prior assumptions into the estimation process [19, 35, 46]. Research has shown that prior assumptions can influence posterior estimates [20, 46–48]. In this study, we explored different prior assumptions for suppressed data and demonstrated model comparison at each level of data granularity. We cannot say which model produces the most valid estimates of suppressed values for several reasons. First, the three prior assumptions selected are for illustrative purposes only, because they are only a subset of all possible prior distributions. Researchers can expand upon our approach by considering more complex prior distributions or incorporating known information about suppressed death counts in choosing prior distributions. Second, the choice of prior distributions is subjective and can vary by analyst. With three levels of data aggregation to choose from, researchers can select the best fitted model based on the most appropriate model performance measures for each aggregate level. In addition, while noninformative priors performed the best at the data element level (elpd_loo results), it tends to overfit the (unsuppressed) data [19, 20, 46, 48]. If researchers are concerned with the quality of unsuppressed data (e.g., measurement error, systematic data collection issues), weakly informative priors might be a better choice in the estimation process [20, 46–48].

In our study, we demonstrated why the imputation results can help inform public health decision-making during a pandemic via the estimation of CDR and ASDR at the county level. Without age-specific COVID-19 deaths, CDR is the primary metric used to understand disparities in deaths from COVID-19 among US counties. However, CDR is influenced by the population’s age composition and might be misleading when comparing populations [41]; for COVID-19, the size of the elderly population is the primary driver of death in many communities. ASDR, which removes the influence of confounding age composition in the population, is the alternative. In our analysis, we found that CDR and ASDR resulted in very different rankings of counties most affected by COVID-19 mortality. If death rate is used as the metric for resource allocation to reduce disparity in death from COVID-19, ASDR is a better metric than CDR because CDR might bias resource allocation toward counties with a larger elderly population. In addition, we found that for COVID-19, ASDR was more highly correlated with SVI than CDR at the county level. This suggests that ASDR better reflects factors not directly correlated with age (e.g., poverty, crowded housing). Furthermore, public health researchers can use the imputation results that reduced the bias from suppressed provisional data to investigate the impact of policies such as non-pharmaceutical interventions and vaccine uptake on COVID-19 deaths in a timely manner. As the pandemic continues, this imputation method can be applied to the provisional death data in 2021 and 2022 to facilitate analysis for public health policies in a rapidly changing pandemic.

Our study is subject to several limitations. First, we did not use a discrete distribution for count data due to the computational constraint of Stan [32]. Nonetheless, the gamma distribution can be used to model a right-skewed distribution such as COVID-19 mortality. For future studies, researchers can change the model specifications to distributions that are more appropriate for count data by adapting from the modeling process provided in this study. Second, although variables such as health insurance coverage, income level, and minority status might improve the model fit, we did not include them to reduce computational burden. The model presented in this study cost about two days’ worth of computational effort to fit and test; additional variables could make the model more computationally intensive. Third, despite outperforming estimates with no imputation, our models generally overestimated all age groups except for ages 18–29 years. This overestimation issue could be addressed by adding more detailed information (e.g., state- or locality-level quarterly data by age group) [48, 49]. In the process of model building, we noticed that as age-specific annual COVID-19 deaths at the state/locality level were added to the likelihood, overestimation was substantially reduced. However, detailed data are more likely to suffer from data suppression. We conducted analyses among different prior distributions only for suppressed data. However, more informative prior distributions could be used in the estimation of fixed and random effects in the model. Fourth, although the structure of our multilevel model accounted for the correlation among the counties within a state, this model did not account for the spatial relationship among adjacent counties [14, 15, 50]. Future studies can explore models such as conditional autoregressive models, to capture the correlated mortality rate through spatial relationships. Finally, results from this study are only applicable to the release of the provisional COVID-19 mortality data. As the final 2020 mortality data are now available, researchers can apply to access restricted-use data that do not have data suppression through CDC WONDER [51, 52]. Nonetheless, this method is still applicable to the nonrestricted-use final COVID-19 mortality data publicly available through CDC WONDER because the nonrestricted-use data still contain data suppression. In addition, this method can be applicable to address the suppressed data in future provisional data (e.g., 2021, 2022), mortality for causes beyond COVID-19, and other types of publicly available datasets.

## Conclusion

Data suppression might limit the analytical use of publicly available datasets, especially when the proportion of data suppressed is high. We demonstrated a flexible Bayesian approach that can model the data generating processes and integrate different levels of data granularity to impute suppressed COVID-19 death counts by age group, quarter, and county for 2020. This imputation approach can help uncover age-specific COVID-19 deaths in the provisional data and facilitate further analyses, such as estimating COVID-19 ASDR by county. Compared with CDR, use of ASDR that can only be estimated from the imputation results might lead to different resource allocation decisions intended to reduce disparity in deaths from COVID-19 among US communities.

## Supporting information

S1 File Supplemental methods. (DOCX)

S2 File Supplemental results. (DOCX)

S1 Table A sample dataset of COVID-19 deaths by quarter and age in Bronx county, New York in 2020. (DOCX)

S2 Table Model performance across three model assumptions of prior distribution for suppressed data. (DOCX)

S3 Table Top 20 counties most affected by COVID-19–associated deaths in 2020 based on the age-standardized death rate calculated from imputation models M2 and M3. (DOCX)

S1 Fig Distribution of suppressed and unsuppressed COVID-19 deaths by quarter in the county-level provisional COVID-19 deaths dataset and the number of counties that had at least one COVID-19 death by quarter in 2020. (TIFF)

S2 Fig Distribution of suppressed and unsuppressed COVID-19 deaths by quarter, age, and urban-rural code in the county-level provisional COVID-19 deaths dataset. (TIFF)

S3 Fig Distributions of counties with 0, 1, 2, …, ≥20 COVID-19 deaths with simulated data from the fitted Bayesian model and observed data by three model assumptions of the prior distribution for suppressed death counts. (TIFF)

S4 Fig Correlation between predicted and observed annual COVID-19 deaths by age group and state/locality. (TIFF)

S5 Fig Number of COVID-19 deaths by age group and state/locality between data aggregated from imputation results based on M1 and from state-/locality-level provisional dataset. (TIFF)

S6 Fig The correlation between county Social Vulnerability Index and crude death rates from USAFacts (ρ = 0.1880) or age-standardized death rates from imputation results (ρ = 0.3249 for M1, ρ = 0.3208 for M2, ρ = 0.3212 for M3). (TIFF)
