# Supplementary material for: Better data for decision-making through Bayesian imputation of suppressed provisional COVID-19 death counts
Source: PLoS One. 2023 Aug 3;18(8):e0288961. doi: 10.1371/journal.pone.0288961 (PMC10399909; doi:10.1371/journal.pone.0288961)
Supplement: S1 File — (DOCX) [file pone.0288961.s001.docx]

**Supplemental methods**

1. **Imputation for annual COVID-19 death counts at the state or locality level in 2020**

To impute the suppressed COVID-19 death counts at the state/locality level, we first considered the dataset with all age groups, including the age group 0–17 years. After we completed the imputation process described below, we removed the death counts for age group 0–17 years in the final state-/locality-level dataset used for the imputation model. The state-/locality-level data contained 416 data rows (8 age groups x 52 states and localities) in which 68 age-specific annual COVID-19 death counts were suppressed. The suppressed annual COVID-19 death counts by age group and state/locality are presented in Table A1. We imputed these suppressed annual COVID-19 deaths at the state/locality level using the following steps. First, we calculated the difference between the reported total annual COVID-19 death counts in a state/locality and the aggregate annual COVID-19 death counts among all age groups that had unsuppressed annual COVID-19 deaths reported in the state/locality. The difference was the total COVID-19 death counts for the age groups that had suppressed COVID-19 death counts in the state/locality. Second, we obtained the values of 34 suppressed annual COVID-19 death counts because these data elements were the single suppressed COVID-19 death count among all age groups in the state/locality. The remaining 34 suppressed data elements were distributed among 14 states and localities; that is, each of the 14 states/localities had suppressed annual COVID-19 death counts for more than one age group. Third, to address the suppressed data elements in these 14 states and localities, as demonstrated in the example that follows, we used the age distributions of total annual death counts in these states/localities that were also reported in the dataset to impute these suppressed records. The information of total annual death counts was used to infer these suppressed records because (a) total annual death counts by age group were all present at the state/locality level; and (b) the age distribution of total annual death counts was highly correlated with the age distribution of COVID-19 death counts at the state or locality level (ρ = 0.9770). For each state/locality that had suppressed annual COVID-19 death counts for more than one age group, we calculated the age distribution of the total annual death counts among the corresponding age groups that had suppressed annual COVID-19 death counts. We then reallocated the total COVID-19 death counts for all suppressed age groups in the state/locality by the age distribution from the annual death counts.

**Table A1. Distribution of suppressed annual COVID-19 death counts by age group and state/locality based on the 2020 NCHS aggregate-level provisional COVID-19 death data.**

|  | **Age Groups** | | | | |  |
| --- | --- | --- | --- | --- | --- | --- |
| **State/locality** | **0–17** | **18–29** | **30–39** | **40–49** | **50–64** | **Number of age groups with suppressed COVID-19 deaths in the corresponding state/locality** |
| Alabama | 1 |  |  |  |  | 1 |
| Arizona | 1 |  |  |  |  | 1 |
| Arkansas | 1 |  |  |  |  | 1 |
| Colorado | 1 |  |  |  |  | 1 |
| Florida | 1 |  |  |  |  | 1 |
| Georgia | 1 |  |  |  |  | 1 |
| Illinois | 1 |  |  |  |  | 1 |
| Indiana | 1 |  |  |  |  | 1 |
| Iowa | 1 |  |  |  |  | 1 |
| Louisiana | 1 |  |  |  |  | 1 |
| Maryland | 1 |  |  |  |  | 1 |
| Massachusetts | 1 |  |  |  |  | 1 |
| Michigan | 1 |  |  |  |  | 1 |
| Minnesota | 1 |  |  |  |  | 1 |
| Mississippi | 1 |  |  |  |  | 1 |
| Missouri | 1 |  |  |  |  | 1 |
| Montana |  | 1 |  |  |  | 1 |
| Nebraska | 1 |  |  |  |  | 1 |
| Nevada | 1 |  |  |  |  | 1 |
| New Jersey | 1 |  |  |  |  | 1 |
| New Mexico | 1 |  |  |  |  | 1 |
| New York | 1 |  |  |  |  | 1 |
| North Carolina | 1 |  |  |  |  | 1 |
| Ohio | 1 |  |  |  |  | 1 |
| Oklahoma | 1 |  |  |  |  | 1 |
| Oregon |  | 1 |  |  |  | 1 |
| Pennsylvania | 1 |  |  |  |  | 1 |
| South Carolina | 1 |  |  |  |  | 1 |
| South Dakota |  | 1 |  |  |  | 1 |
| Tennessee | 1 |  |  |  |  | 1 |
| Utah | 1 |  |  |  |  | 1 |
| Virginia | 1 |  |  |  |  | 1 |
| Washington | 1 |  |  |  |  | 1 |
| Wisconsin | 1 |  |  |  |  | 1 |
| Alaska |  | 1 | 1 |  |  | 2 |
| Connecticut | 1 | 1 |  |  |  | 2 |
| Delaware | 1 | 1 |  |  |  | 2 |
| District of Columbia | 1 | 1 |  |  |  | 2 |
| Hawaii |  | 1 | 1 |  |  | 2 |
| Idaho |  | 1 | 1 |  |  | 2 |
| Kentucky | 1 | 1 |  |  |  | 2 |
| North Dakota | 1 | 1 |  |  |  | 2 |
| Maine |  | 1 | 1 | 1 |  | 3 |
| New Hampshire |  | 1 | 1 | 1 |  | 3 |
| Rhode Island | 1 | 1 | 1 |  |  | 3 |
| Vermont |  |  | 1 | 1 | 1 | 3 |
| West Virginia | 1 | 1 | 1 |  |  | 3 |
| Wyoming |  | 1 | 1 | 1 |  | 3 |
| **Total** | 38 | 16 | 9 | 4 | 1 | 68 |
| *Note*: Value 1 represents a suppressed COVID-19 death count in the age group in the state/locality in 2020, meaning that the COVID-19 death count was between 1 and 9. Blank cells indicate that the COVID-19 death count in the age group in the state/locality was not suppressed. | | | | | | |

We present New Hampshire as an example to describe the imputation process. In New Hampshire, the annual COVID-19 death counts for age groups 18–29, 30–39, and 40–49 years were suppressed in the dataset. Because the number of COVID-19 deaths for the entire state was 1,335 and the number of unsuppressed COVID-19 deaths was 1,321, we could reallocate the remaining 14 COVID-19 deaths (1,335 – 1,321 = 14) among age groups 18–29, 30–39, and 40–49 years. Among these three age groups, the total annual deaths (i.e., deaths from any causes) were 270 deaths (21% = 270/1,273) for those aged 18–29 years, 424 deaths (33% = 270/1,273) for those aged 30–39 years, and 579 deaths (46% = 270/1,273) for those aged 40–49 years, respectively. Using the distribution of the total annual deaths among the three age groups, the estimated annual COVID-19 deaths were 3 deaths (= 14 x 21%) for those aged 18–29 years, 5 deaths (= 14 x 33%) for those aged 30–39 years, and 6 deaths (= 14 x 46%) for those aged 40–49 years in New Hampshire.

After conducting the simple imputation for the data at the state/locality level, annual COVID-19 deaths in age group 0–17 years were excluded from the final aggregate-level dataset to be consistent with the county-level dataset.

1. **Model specification of the county-level model**

The logistic regression for zero and positive outcomes for the hurdle gamma model is specified below:

$$\begin{aligned} logit\left( \hat{p}_{aijt} \right)=\tilde{\alpha}_{ij}+\tilde{X}_{aijt}^{T}\tilde{\beta}\#\left( 1 \right) \end{aligned}$$

$$\begin{aligned} \tilde{\alpha}_{ij}=\tilde{\theta}_{j}+\nu_{ij} \#\left( 2 \right) \end{aligned}$$

$$\begin{aligned} \tilde{\alpha}_{j}=\tilde{\theta}_{0}+\tilde{\nu}_{j} \#\left( 3 \right) \end{aligned}$$

$$\begin{aligned} \tilde{\beta}\sim Normal(0, 10)\#\left( 4 \right) \end{aligned}$$

$$\begin{aligned} \tilde{\theta}_{0}, \tilde{\theta}_{j}\sim Normal(0, 1) \#\left( 5 \right) \end{aligned}$$

$$\begin{aligned} v_{ij}, \tilde{v}_{j}\sim studentT\left( 3, 0, 2.5 \right), v_{ij}, \tilde{v}_{j}>0 \#\left( 6 \right) \end{aligned}$$

In equation (1), $\hat{p}_{aijt}$ is the predicted probability of positive death count for age group *a* in county *i* located in state/locality *j* in quarter *t*, $\tilde{\alpha}_{ij}$ represents the intercept and $\tilde{\beta}$ denotes a vector of coefficients corresponding to the covariates in the design matrix $\tilde{X}_{aijt}$. The design matrix $\tilde{X}_{aijt}$ contained urban-rural codes, age groups, quarters, and the interaction terms between age groups and quarters and between quarters and urban-rural codes. We assumed random intercepts at the county level in equation (2) and the state level in equation (3). The prior distributions of coefficients ($\tilde{\beta}$), the county- and state-/locality-level random intercepts ($\tilde{\theta}_{j}$ and $\tilde{\theta}_{0}$), and the county- and state-level error terms ($\nu_{ij}$ and $\tilde{\nu}_{j}$) are specified in equations (4), (5), and (6), respectively.

Regarding the positive outcomes, we followed the model specification with a log-link function expressed below:

$$\begin{aligned} log\left( \hat{\mu}_{aijt} \right)=\alpha_{ij}+X_{ijt}^{T}\beta\#\left( 7 \right) \end{aligned}$$

$$\begin{aligned} \alpha_{ij}=\theta_{j}+u_{ij}\#\left( 8 \right) \end{aligned}$$

$$\begin{aligned} \alpha_{j}=\theta_{0}+\tilde{u}_{j}\#\left( 9 \right) \end{aligned}$$

$$\begin{aligned} \beta\sim Normal(0, 10)\#\left( 10 \right) \end{aligned}$$

$$\begin{aligned} \theta_{0}, \theta_{j}\sim Normal(0, 1) \#\left( 11 \right) \end{aligned}$$

$$\begin{aligned} u_{ij}, \tilde{u}_{j}\sim studentT\left( 3, 0, 2.5 \right), v_{ij}, \tilde{v}_{j}>0 \#\left( 12 \right) \end{aligned}$$

In equation (7), $\hat{\mu}_{aijt}$ is the predicted count given that the outcome is positive for age group *a* in county *i* located in state/locality *j* in quarter *t*; $\alpha_{ij}$ represents the intercept, and $\beta$ denotes a vector of coefficients corresponding to the covariates in the design matrix $X_{ijt}^{T}$. The covariates in the design matrix $X_{ijt}^{T}$ included quarters, age groups, urban-rural codes, and log-transformed population size. The design matrix also included the interaction terms between quarters and age groups, quarters and urban-rural codes, and age groups and urban-rural codes. Like the model specification used in the logistic regression, we assumed random intercepts at the county and state levels in equations (8) and (9), respectively. In equations (4) and (5), the prior distributions of coefficients ($\beta$), the county- and state-level parameters ($\theta_{j}$ and $\theta_{0}$), and the county- and state-level error terms ($u_{ij}$ and $\tilde{u}_{j}$) are specified in equations (10), (11), and (12).

1. **Computer simulations**

The initial values of the parameters for estimation were determined by Stan [1]. After the initial values were determined, we repeated the following steps at each iteration until model convergence.

1. Draw a sample of parameters, including the coefficients in the hurdle model and the suppressed data.
2. Transform the parameter sample to calculate the expected death counts, which is the product in equation (3), for age group *a* in county *i* in quarter *t*.
3. Aggregate the expected death counts over counties and quarters to obtain the age-specific predicted death counts by state and locality (equation 4).
4. Aggregate the expected death counts over counties, states, and quarters to obtain national age-specific predicted death counts at the national level (equation 5).
5. Compute the posterior probability that multiplied the priors of the current parameter sample by the likelihood determined by the estimated quantities at different levels of data modeling generated from the parameter sample set.

**References:**

1. Carpenter B, Gelman A, Hoffman MD, Lee D, Goodrich B, Betancourt M, et al. *Stan* : A Probabilistic Programming Language. J Stat Softw. 2017;76. doi:10.18637/jss.v076.i01
2. **Model performance, comparison, and validation**

We assessed model performance at the national, state or locality, and data element levels for each fitted model. To assess model performance at the national and state/locality levels, we compared the aggregate death counts estimated from the 1,000 imputation datasets for each imputation model with the reported death counts from the aggregate-level provisional data. In the aggregate-level provisional data, all the reported national death counts by each age group were present; the reported state-/locality-level death counts by age group were either present or imputed (S1 File, Supplemental methods 1). At the national level, we estimated the national death counts for all adults aged ≥18 years and for each age group, respectively, using each imputation dataset. The estimated national death counts for all adults aged ≥18 years are defined as the sum of all deaths in each dataset; the national deaths for each age group are the sum of age-specific death counts over all counties and quarters in each dataset. We then estimated percent bias, which measures the percent relative difference of the estimated aggregate death counts to the national death counts by age group and for all adults aged ≥18 years respectively reported from the aggregate-level provisional data [1]. Positive percent bias indicates that the model overestimates deaths; negative percent bias suggests that the model underestimates deaths. At the state or locality level, we estimated the state- or locality-level death counts for each age group for each imputation dataset. The state- or locality-level death counts for each age group are defined as the sum of age-specific death counts over quarters and counties within a state. Root mean squared error (RMSE) was estimated to measure the difference between the estimated and reported state-/locality-level counts from the aggregate-level provisional data [2, 3]. A smaller RMSE suggests a better model fit. The aggregate-level age-specific death counts reported in the aggregate-level provisional data were considered “true” death counts. Therefore, comparing the death estimates from the 1,000 imputation datasets to the true death counts reported at the corresponding aggregate-level provisional data serves as a top-level validation of the imputation results from our models.

At the data element level, we estimated expected log predictive density through leave-one-out cross-validation (elpd_loo) via loo package in R using the provisional data by quarter, county, and age [4]. Elpd_loo approximates the leave-one-out cross-validation, which measures how well a model predicts a data point in the dataset if the model is only fitted to the rest of the data in the dataset by leaving out the specific data point, with faster and stable computation [4, 5]. A model with a larger elpd_loo performs better in predicting new data than a model with a smaller elpd_loo. In addition, the loo package also produces Pareto *k* diagnostics, which can identify the data points that are not well predicted by the model. Because of the algorithm in the loo package, all posterior samples from the Bayesian estimation process were used for estimating elpd_loo. These model performance measures were calculated for each model and were used to compare the model fit among all three models.

**References:**

1. Gupta HV, Sorooshian S, Yapo PO. Status of Automatic Calibration for Hydrologic Models: Comparison with Multilevel Expert Calibration. J Hydrol Eng. 1999;4: 135–143. doi:10.1061/(ASCE)1084-0699(1999)4:2(135)
2. Sun H, Yang Y, Wu R, Gui D, Xue J, Liu Y, et al. Improving Estimation of Cropland Evapotranspiration by the Bayesian Model Averaging Method with Surface Energy Balance Models. Atmosphere. 2019;10: 188. doi:10.3390/atmos10040188
3. Fu YH, Campioli M, Van Oijen M, Deckmyn G, Janssens IA. Bayesian comparison of six different temperature-based budburst models for four temperate tree species. Ecol Model. 2012;230: 92–100. doi:10.1016/j.ecolmodel.2012.01.010
4. Vehtari A, Gelman A, Gabry J. Practical Bayesian model evaluation using leave-one-out cross-validation and WAIC. Stat Comput. 2017;27: 1413–1432. doi:10.1007/s11222-016-9696-4
5. Kennedy L, Simpson D, Gelman A. The Experiment is just as Important as the Likelihood in Understanding the Prior: a Cautionary Note on Robust Cognitive Modeling. Comput Brain Behav. 2019;2: 210–217. doi:10.1007/s42113-019-00051-0
