# Supplementary material for: Better data for decision-making through Bayesian imputation of suppressed provisional COVID-19 death counts
Source: PLoS One. 2023 Aug 3;18(8):e0288961. doi: 10.1371/journal.pone.0288961 (PMC10399909; doi:10.1371/journal.pone.0288961)
Supplement: S2 File — (DOCX) [file pone.0288961.s002.docx]

**Supplemental results**

1. **Model diagnostics**

Model convergence was supported by the traceplots (Fig A1) and diagnostic statistics (maximum $\hat{R}$ close to 1 and the percent of effective sample size per iteration >10% among all parameters) for all three models. The posterior predictive results indicated that all models replicated the observed data well (S3 Fig).

1. Traceplots of M1: noninformative priors for all age groups.


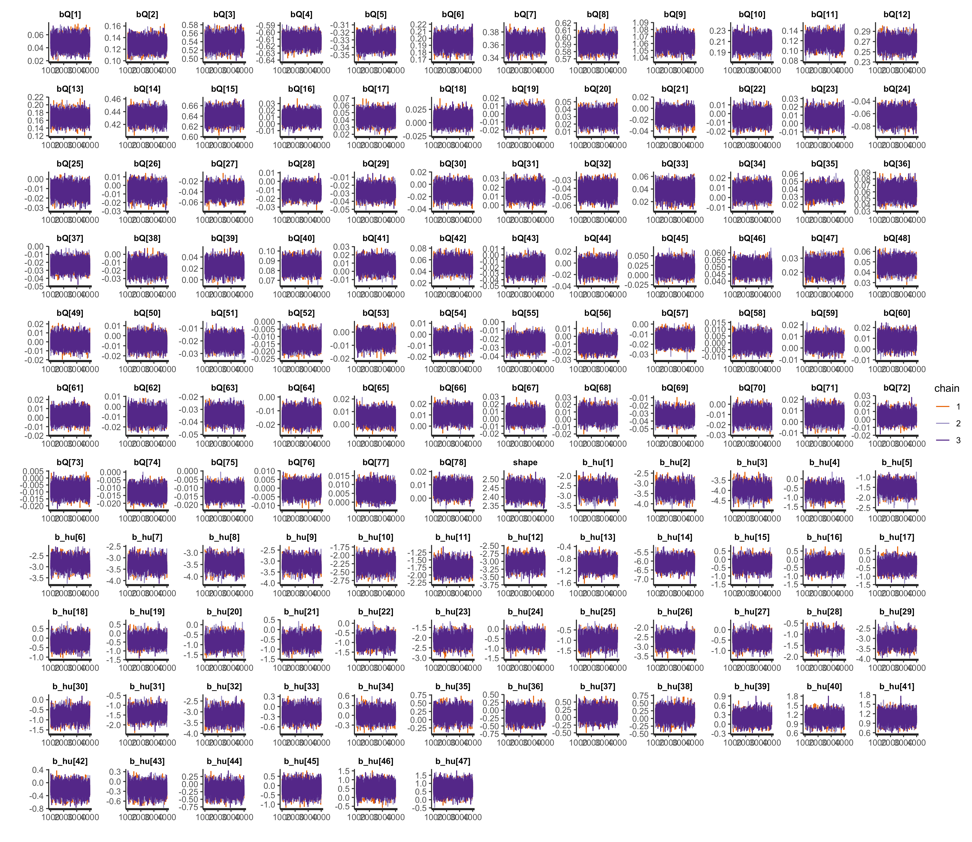


1.
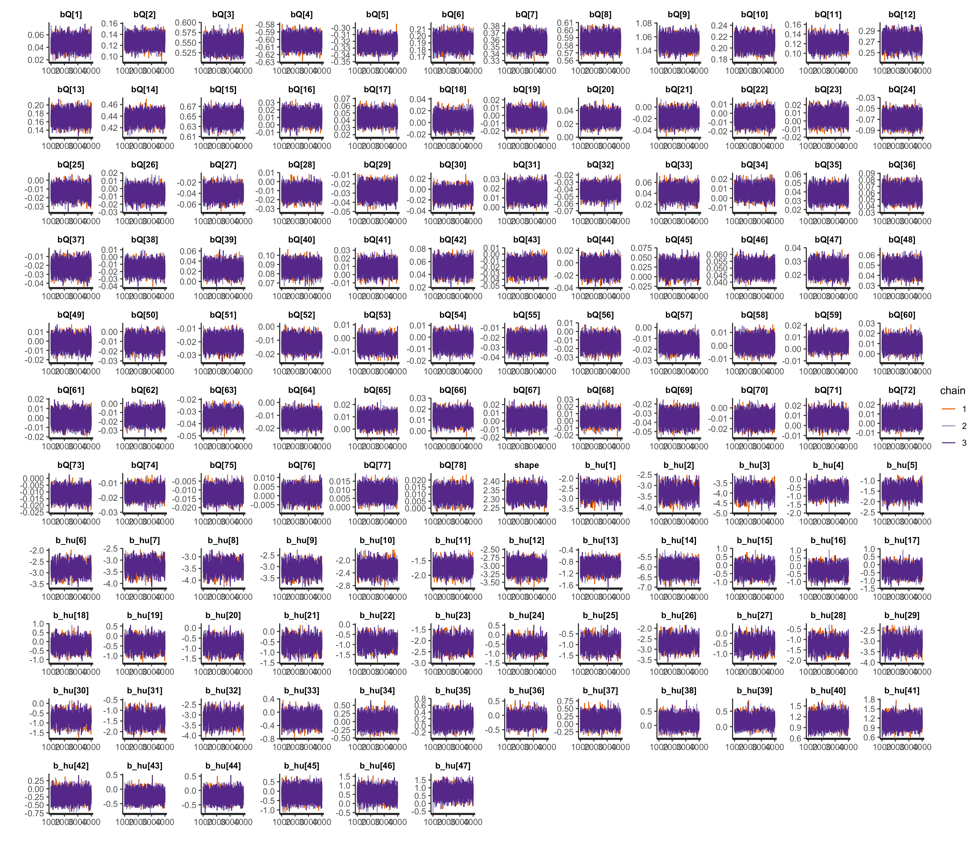
Traceplots of M2: same weakly informative priors for all age groups.
2. Traceplots of M3: different weakly informative priors by age groups 18–49 years and ≥50 years.


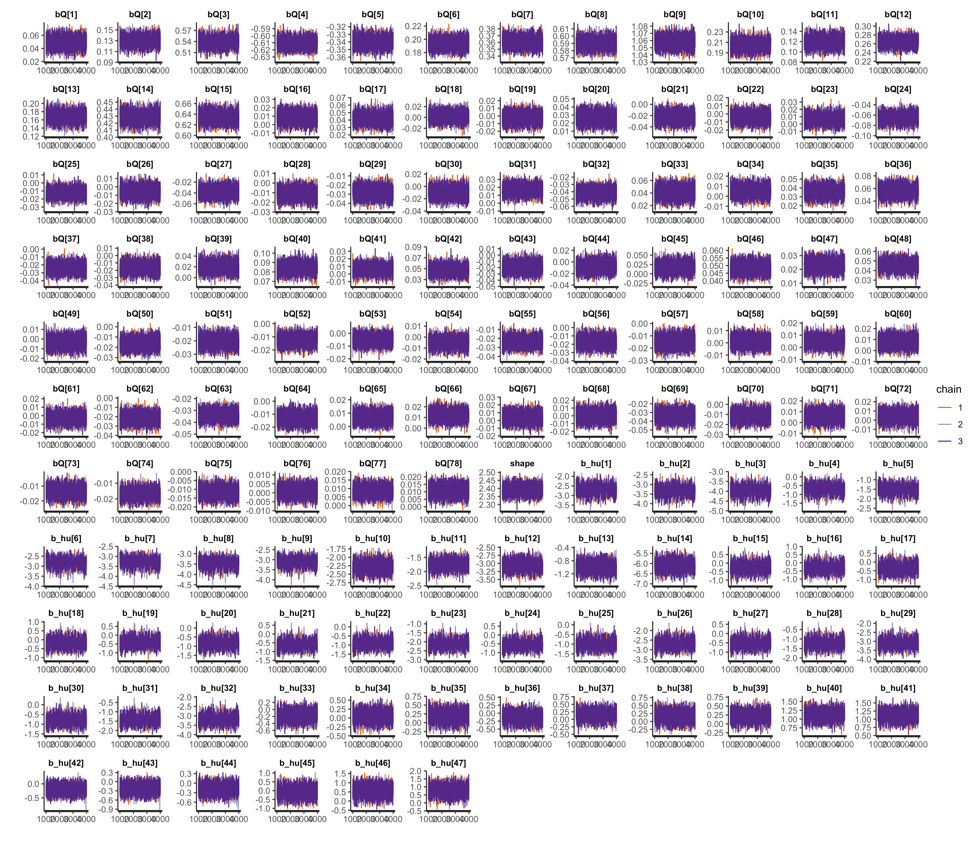


**Fig A1. Traceplots of fixed-effects parameters for each model assumption of the prior distribution for suppressed data.**

1. **External validation by comparison to public measures**

Although USAFacts generally underestimate the true COVID-19 death counts (8.7% lower than the national death counts from the provisional data), the data from USAFacts are publicly available without data suppression. We assumed that the COVID-19 death counts at the county level from USAFacts preserve the geospatial distribution of the death counts among US counties. Hence, we examined the correlation coefficients between the results from the imputation model and the data from USAFacts. The aggregate predicted death counts at the county level were highly correlated with the death counts reported in USAFacts ([ρ = 0.9875; 95% CI: 0.9874, 0.9876] for M1; [ρ = 0.9876, 95% CI: 0.9874, 0.9877] for M2; and [ρ = 0.9876, 95% CI: 0.9875, 0.9877] for M3). In general, the aggregate death counts at the county level from the imputation results were higher than the data from USAFacts, varying with the urbanicity (Table A2). The percent difference between the predicted and reported county-level deaths was the lowest, with the smallest variation, among large central metro counties but the largest, with the widest variation, among noncore counties across all three models.

**Table A2. Percent difference between the predicted COVID-19 deaths aggregate from the imputation results and reported county-level COVID-19 deaths from USAFacts.**

|  | **M1:  Noninformative prior for all age groups** | | **M2:  Same weakly informative prior for all age groups** | | **M3:  Different weakly informative prior by age groups 18–49 years and ≥50 years** | |
| --- | --- | --- | --- | --- | --- | --- |
| Urban-rural code | Median | Standard deviation | Median | Standard deviation | Median | Standard deviation |
| Large central metro | 6.3% | 26.8% | 6.0% | 26.6% | 5.8% | 26.6% |
| Large fringe metro | 23.9% | 141.2% | 20.8% | 133.3% | 23.0% | 139.9% |
| Medium metro | 21.3% | 113.2% | 19.3% | 108.6% | 20.4% | 112.6% |
| Small metro | 24.6% | 141.7% | 21.7% | 135.5% | 23.6% | 142.8% |
| Micropolitan | 31.9% | 155.0% | 27.5% | 149.5% | 30.7% | 153.9% |
| Noncore | 45.5% | 214.4% | 37.5% | 200.2% | 43.1% | 211.1% |
