# Supplementary material for: Better data for decision-making through Bayesian imputation of suppressed provisional COVID-19 death counts
Source: PLoS One. 2023 Aug 3;18(8):e0288961. doi: 10.1371/journal.pone.0288961 (PMC10399909; doi:10.1371/journal.pone.0288961)
Supplement: S1 Table — (DOCX) [file pone.0288961.s003.docx]

**S1 Table. A sample dataset of COVID-19 deaths by quarter and age in Bronx County, New York in 2020.** Suppressed data cells (counts between 1 and 9) are left blank.

| **Quarter** | **State** | **County** | **FIPS Code** | **Urban-Rural Code** | **Age Group** | **COVID-19 Deaths** |
| --- | --- | --- | --- | --- | --- | --- |
| … | … | … | … | … | … | … |
| 1 | NY | Bronx County | 36005 | Large central metro | 18–29 years |  |
| 2 | NY | Bronx County | 36005 | Large central metro | 18–29 years | 16 |
| 3 | NY | Bronx County | 36005 | Large central metro | 18–29 years |  |
| 4 | NY | Bronx County | 36005 | Large central metro | 18–29 years |  |
| 1 | NY | Bronx County | 36005 | Large central metro | 30–39 years |  |
| 2 | NY | Bronx County | 36005 | Large central metro | 30–39 years | 66 |
| 3 | NY | Bronx County | 36005 | Large central metro | 30–39 years |  |
| 4 | NY | Bronx County | 36005 | Large central metro | 30–39 years |  |
| 1 | NY | Bronx County | 36005 | Large central metro | 40–49 years | 29 |
| 2 | NY | Bronx County | 36005 | Large central metro | 40–49 years | 168 |
| 3 | NY | Bronx County | 36005 | Large central metro | 40–49 years |  |
| 4 | NY | Bronx County | 36005 | Large central metro | 40–49 years |  |
| 1 | NY | Bronx County | 36005 | Large central metro | 50–64 years | 111 |
| 2 | NY | Bronx County | 36005 | Large central metro | 50–64 years | 824 |
| 3 | NY | Bronx County | 36005 | Large central metro | 50–64 years | 18 |
| 4 | NY | Bronx County | 36005 | Large central metro | 50–64 years | 42 |
| 1 | NY | Bronx County | 36005 | Large central metro | 65–74 years | 102 |
| 2 | NY | Bronx County | 36005 | Large central metro | 65–74 years | 976 |
| 3 | NY | Bronx County | 36005 | Large central metro | 65–74 years | 16 |
| 4 | NY | Bronx County | 36005 | Large central metro | 65–74 years | 54 |
| 1 | NY | Bronx County | 36005 | Large central metro | 75–84 years | 108 |
| 2 | NY | Bronx County | 36005 | Large central metro | 75–84 years | 1051 |
| 3 | NY | Bronx County | 36005 | Large central metro | 75–84 years | 20 |
| 4 | NY | Bronx County | 36005 | Large central metro | 75–84 years | 57 |
| 1 | NY | Bronx County | 36005 | Large central metro | 85 years and over | 79 |
| 2 | NY | Bronx County | 36005 | Large central metro | 85 years and over | 779 |
| 3 | NY | Bronx County | 36005 | Large central metro | 85 years and over | 30 |
| 4 | NY | Bronx County | 36005 | Large central metro | 85 years and over | 43 |
| … | … | … | … | … | … | … |
