# Supplementary material for: Better data for decision-making through Bayesian imputation of suppressed provisional COVID-19 death counts
Source: PLoS One. 2023 Aug 3;18(8):e0288961. doi: 10.1371/journal.pone.0288961 (PMC10399909; doi:10.1371/journal.pone.0288961)
Supplement: S2 Table — (DOCX) [file pone.0288961.s004.docx]

**S2 Table. Model performance across three model assumptions of prior distribution for suppressed data.**

|  |  | **Different Assumptions of Prior Distribution for Suppressed Data** | | | | | | | | | |
| --- | --- | --- | --- | --- | --- | --- | --- | --- | --- | --- | --- |
| **Model Performance** |  | **M1:  Noninformative prior for all age groups** | | | **M2:  Same weakly informative prior for all age groups** | | | **M3:  Different weakly informative prior by age groups 18-49 years and ≥50 years** | | |  |
| (A) At the state/locality level | | |  |  |  |  |  |  |  |  |  |
|  |  | Mean | Standard deviation |  | Mean | Standard deviation |  | Mean | Standard deviation |  |  |
| RMSE^†^ |  | 84.08 | 1.86 |  | 68.03 | 1.58 |  | 78.91 | 1.76 |  |  |
|  |  |  |  |  |  |  |  |  |  |  |  |
| (B) At the county level |  |  |  |  |  |  |  |  |  |  |  |
| *Leave-one-out (LOO) estimates* | | | |  |  |  |  |  |  |  |  |
|  |  | Mean | Standard error |  | Mean | Standard error |  | Mean | Standard error |  |  |
| elpd_loo^*^ |  | -43,951.5 | 362.8 |  | -44,107.8 | 364.4 |  | -43,974.2 | 363.6 |  |  |
| p_loo^§^ |  | 1,885.5 | 21.7 |  | 1,898.2 | 22 |  | 1,877.9 | 21.6 |  |  |
| looic^††^ |  | 87,903.0 | 725.6 |  | 88,215.6 | 728.9 |  | 87,948.3 | 727.2 |  |  |
|  |  |  |  |  |  |  |  |  |  |  |  |
| *Pareto k diagnostic values* | | |  |  |  |  |  |  |  |  |  |
|  |  | Count | % | Min | Count | % | Min | Count | % | Min |  |
| (-Inf, 0.5] (good) |  | 64,381 | 99.8% | 903 | 64,390 | 99.8% | 718 | 64,396 | 99.8% | 898 |  |
| (0.5, 0.7] (okay) |  | 112 | 0.2% | 182 | 100 | 0.2% | 279 | 99 | 0.2% | 135 |  |
| (0.7, 1] (bad) |  | 18 | 0.0% | 37 | 21 | 0.0% | 58 | 16 | 0.0% | 39 |  |
| (1, Inf) (very bad) |  | 0 | 0.0% | — | 0 | 0.0% | — | 0 | 0.0% | — |  |
| ^†^Root mean squared error (RMSE) calculated from 1,000 imputation sample sets. ^*^Leave-one-out expected log pointwise predictive density.  ^§^Effective number of parameters. ^††^Leave-one-out information criteria. | | | | | | | | | | | |
