# Supplementary material for: Better data for decision-making through Bayesian imputation of suppressed provisional COVID-19 death counts
Source: PLoS One. 2023 Aug 3;18(8):e0288961. doi: 10.1371/journal.pone.0288961 (PMC10399909; doi:10.1371/journal.pone.0288961)
Supplement: S3 Table — (DOCX) [file pone.0288961.s005.docx]

**S3 Table. Top 20 counties that were most affected by COVID-19–associated deaths in 2020 based on the age-standardized death rate calculated from the imputation models M2 and M3.**

| **M2: Same Weakly Informative Prior for All Age Groups** | | | | **M3: Different Weakly Informative Prior by Age Groups 18–49 Years and ≥50 Years** | | | |
| --- | --- | --- | --- | --- | --- | --- | --- |
| County, state  (FIPS code) | Urban-rural code | Social Vulnerability Index (SVI) | Age-standardized COVID-19 death rate per 100,000 population  [95% CI] | County, state  (FIPS code) | Urban-rural code | Social Vulnerability Index (SVI) | Age-standardized COVID-19 death rate per 100,000 population  [95% CI] |
| Buffalo County, SD (46017) | Noncore | 97.10 | 1,587.89 [1,091.86, 2,415.89] | Buffalo County, SD (46017) | Noncore | 97.10 | 1,645.06 [1,091.86, 2,491.16] |
| Armstrong County, TX (48011) | Medium metro | 15.99 | 941.35 [552.90, 1,483.45] | Armstrong County, TX (48011) | Medium metro | 15.99 | 1,004.00 [587.62, 1,546.11] |
| Issaquena County, MS (28055) | Noncore | 95.10 | 909.85 [570.23, 1,484.19] | Issaquena County, MS (28055) | Noncore | 95.10 | 938.34 [570.23, 1,577.55] |
| Martin County, TX (48317) | Small metro | 42.39 | 772.22 [443.22, 1,209.73] | Martin County, TX (48317) | Small metro | 42.39 | 821.67 [481.81, 1,217.76] |
| Cottle County, TX (48101) | Noncore | 93.63 | 727.10 [414.23, 1,209.16] | Cottle County, TX (48101) | Noncore | 93.63 | 754.84 [414.23, 1,265.91] |
| Benson County, ND (38005) | Noncore | 84.33 | 707.48 [490.73, 962.11] | Benson County, ND (38005) | Noncore | 84.33 | 734.37 [508.46, 985.00] |
| McKinley County, NM (35031) | Micropolitan | 98.89 | 705.43 [687.84, 725.20] | McKinley County, NM (35031) | Micropolitan | 98.89 | 703.20 [687.27, 723.79] |
| Floyd County, TX (48153) | Noncore | 82.99 | 685.05 [516.80, 882.30] | Floyd County, TX (48153) | Noncore | 82.99 | 700.48 [525.13, 905.45] |
| Corson County, SD (46031) | Noncore | 83.95 | 665.74 [363.09, 1,063.36] | Corson County, SD (46031) | Noncore | 83.95 | 693.87 [404.83, 1,080.41] |
| Cochran County, TX (48079) | Noncore | 86.08 | 645.41 [323.55, 1,067.20] | Cochran County, TX (48079) | Noncore | 86.08 | 681.31 [346.35, 1,121.57] |
| Maverick County, TX (48323) | Micropolitan | 97.42 | 630.48 [600.17, 663.74] | Dewey County, SD (46041) | Noncore | 88.31 | 652.22 [377.07, 1,037.73] |
| Dewey County, SD (46041) | Noncore | 88.31 | 618.79 [363.72, 972.45] | Sherman County, TX (48421) | Noncore | 46.11 | 641.06 [323.97, 1,091.95] |
| Sherman County, TX (48421) | Noncore | 46.11 | 601.07 [308.72, 1,024.52] | Maverick County, TX (48323) | Micropolitan | 97.42 | 628.78 [598.04, 660.07] |
| Oldham County, TX (48359) | Medium metro | 43.22 | 600.01 [197.37, 924.17] | Oldham County, TX (48359) | Medium metro | 43.22 | 614 [197.37, 924.17] |
| Hamlin County, SD (46057) | Noncore | 15.86 | 599.37 [536.18, 695.69] | Ziebach County, SD (46137) | Noncore | 95.38 | 611.75 [359.69, 1,059.60] |
| Ziebach County, SD (46137) | Noncore | 95.38 | 584.77 [359.69, 958.73] | Hamlin County, SD (46057) | Noncore | 15.86 | 604.99 [536.18, 712.96] |
| Big Horn County, MT (30003) | Noncore | 82.74 | 572.30 [444.96, 728.05] | Todd County, SD (46121) | Noncore | 95.64 | 598.75 [375.65, 880.88] |
| Todd County, SD (46121) | Noncore | 95.64 | 570.47 [368.89, 836.02] | Dallam County, TX (48111) | Noncore | 55.03 | 592.10 [361.15, 881.72] |
| Starr County, TX (48427) | Micropolitan | 98.15 | 568.77 [542.09, 598.88] | Big Horn County, MT (30003) | Noncore | 82.74 | 591.33 [459.58, 744.70] |
| Neshoba County, MS (28099) | Noncore | 96.85 | 566.21 [514.79, 615.44] | Culberson County, TX (48109) | Noncore | 99.71 | 588.05 [334.89, 1,011.40] |
|  |  | **Average SVI among top 20 counties** |  |  |  | **Average SVI among top 20 counties** |  |
|  |  | **77.01** |  |  |  | **74.99** |  |
