# Supplementary figures and images for: Better data for decision-making through Bayesian imputation of suppressed provisional COVID-19 death counts
Source: PLoS One. 2023 Aug 3;18(8):e0288961. doi: 10.1371/journal.pone.0288961 (PMC10399909; doi:10.1371/journal.pone.0288961)

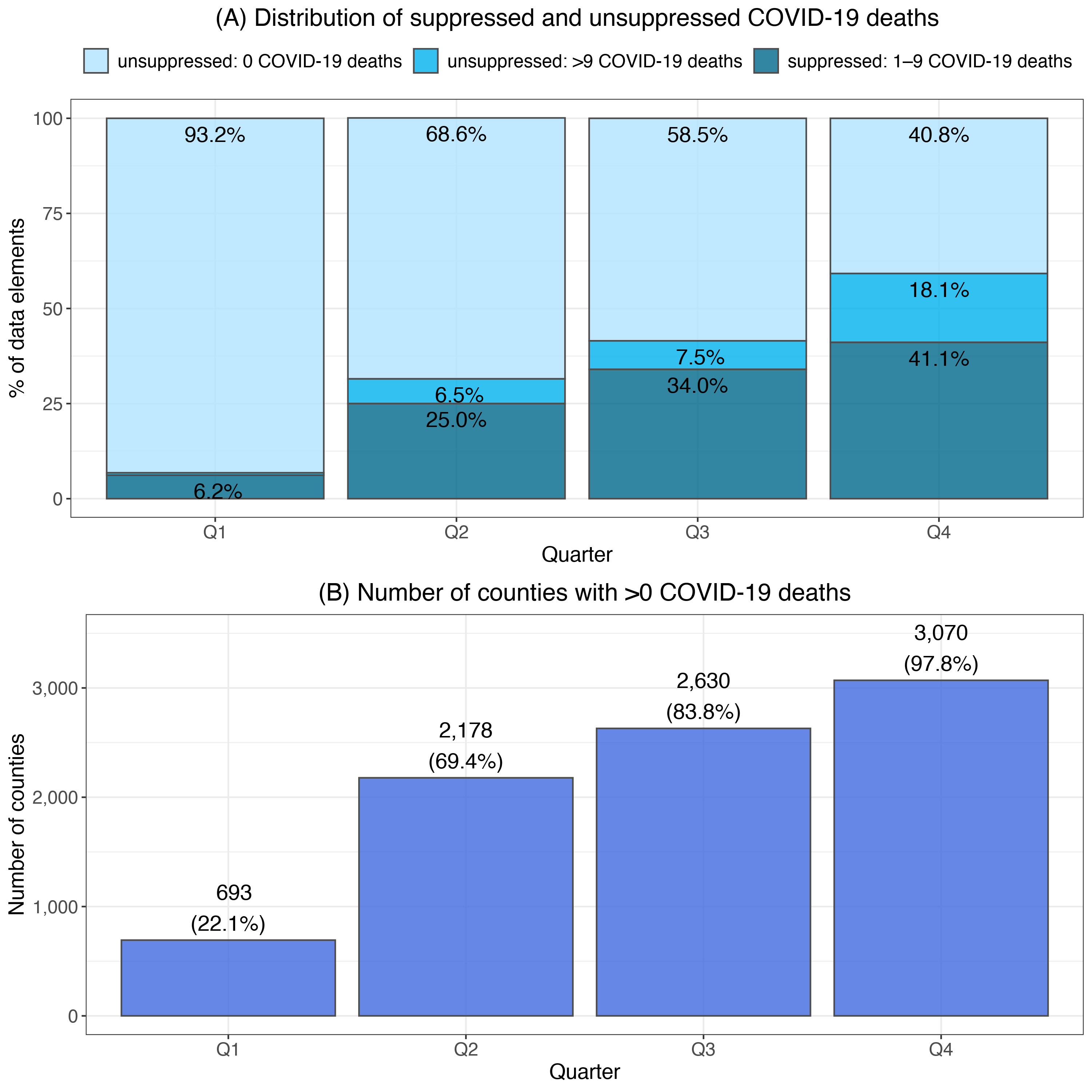

Supplement: S1 Fig — (TIFF) [file pone.0288961.s006.tiff]

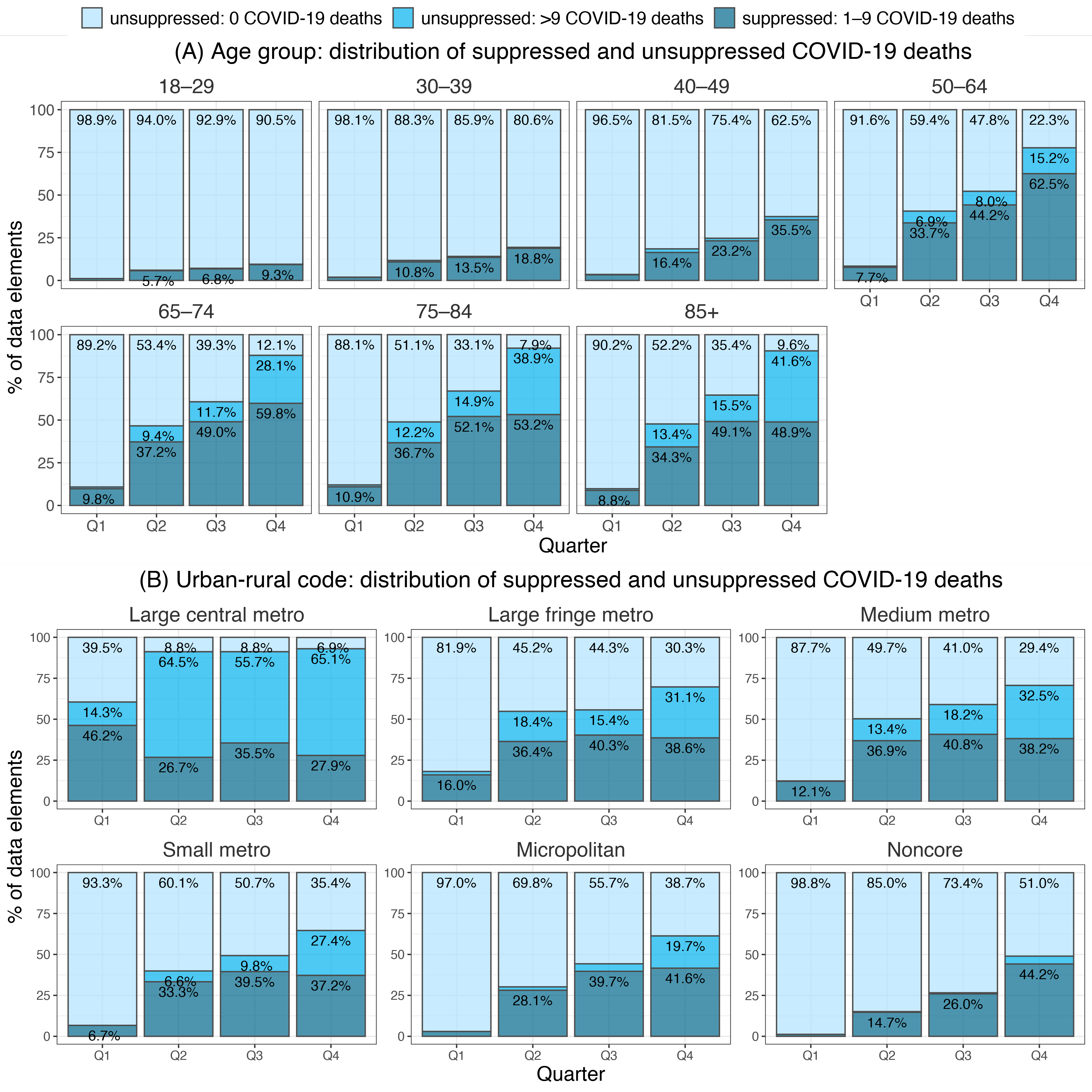

Supplement: S2 Fig — (TIFF) [file pone.0288961.s007.tiff]

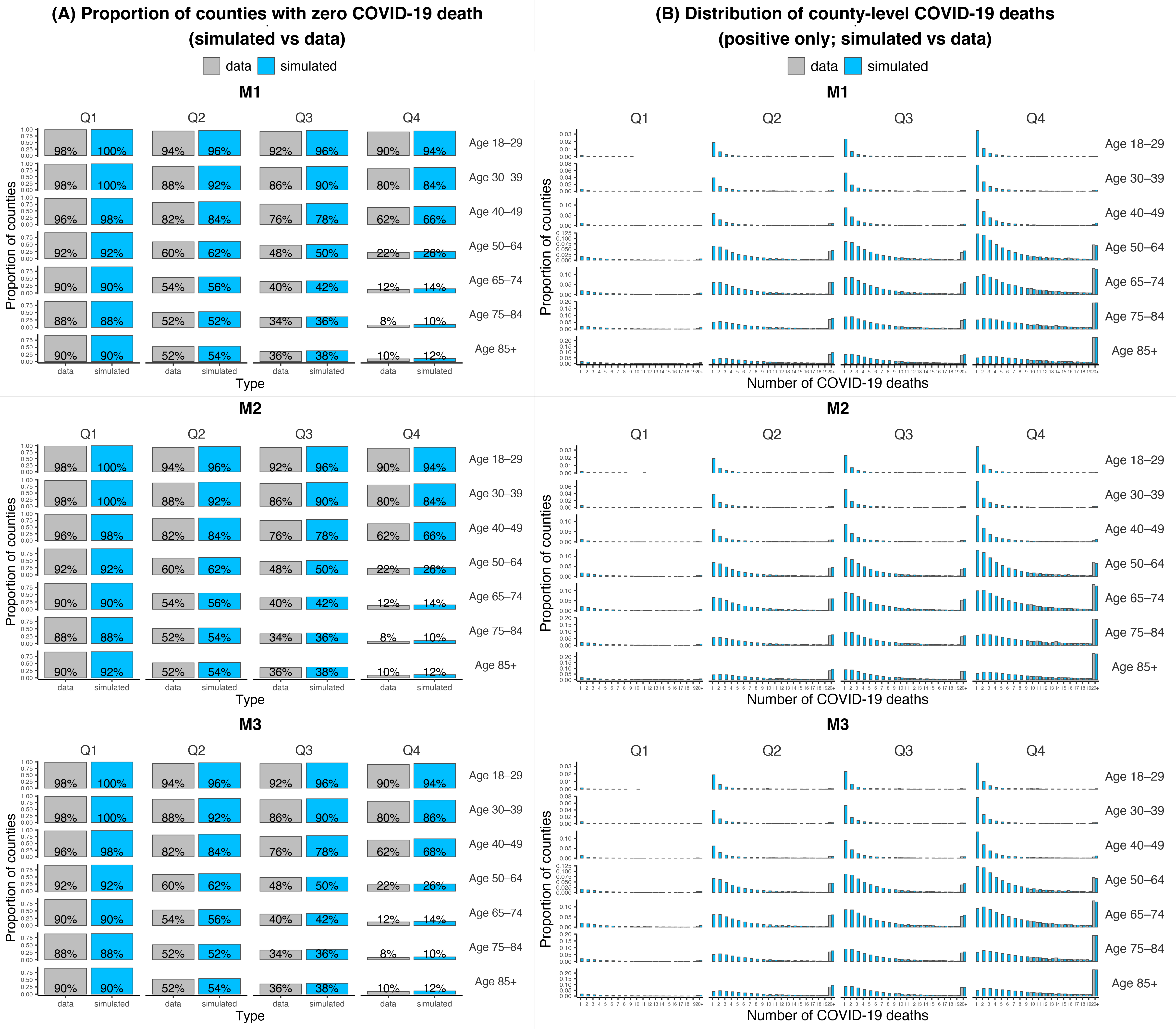

Supplement: S3 Fig — (TIFF) [file pone.0288961.s008.tiff]

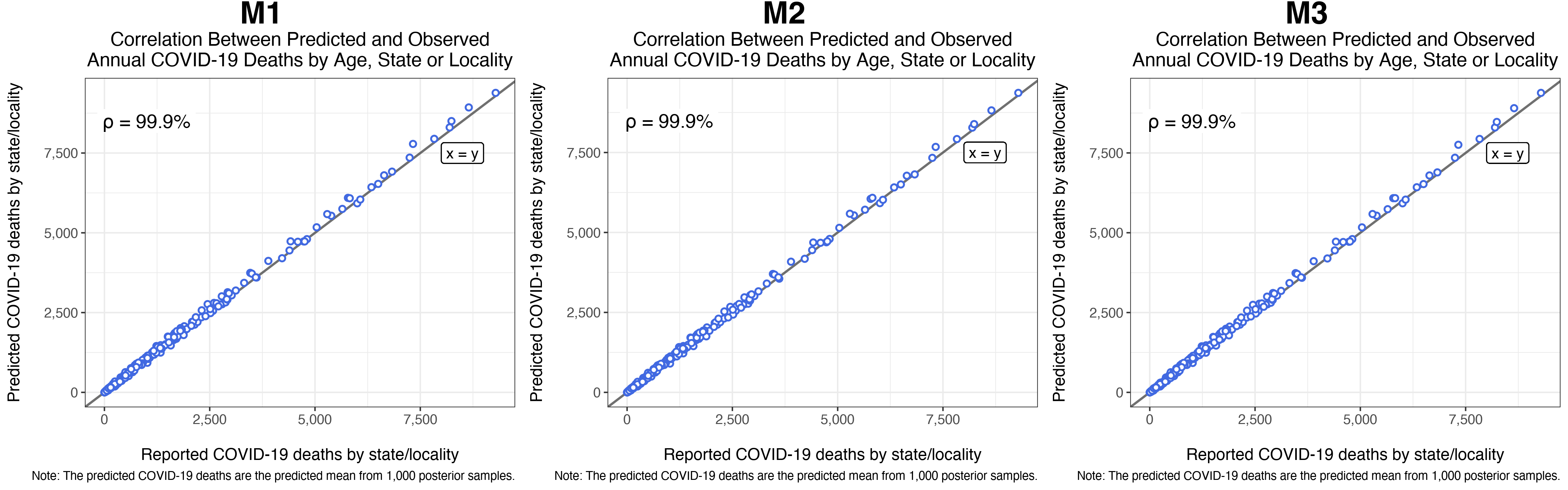

Supplement: S4 Fig — (TIFF) [file pone.0288961.s009.tiff]

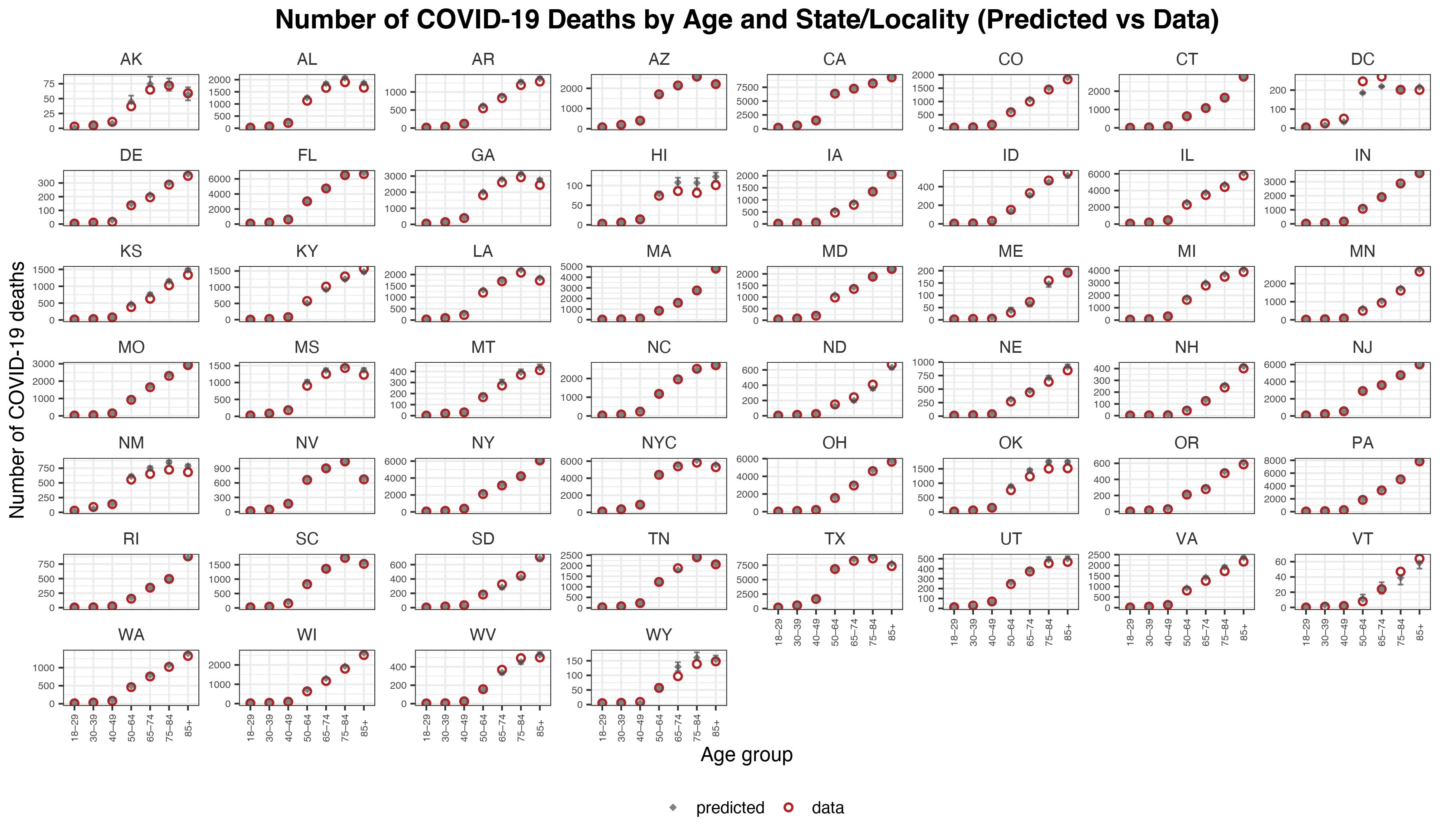

Supplement: S5 Fig — (TIFF) [file pone.0288961.s010.tiff]

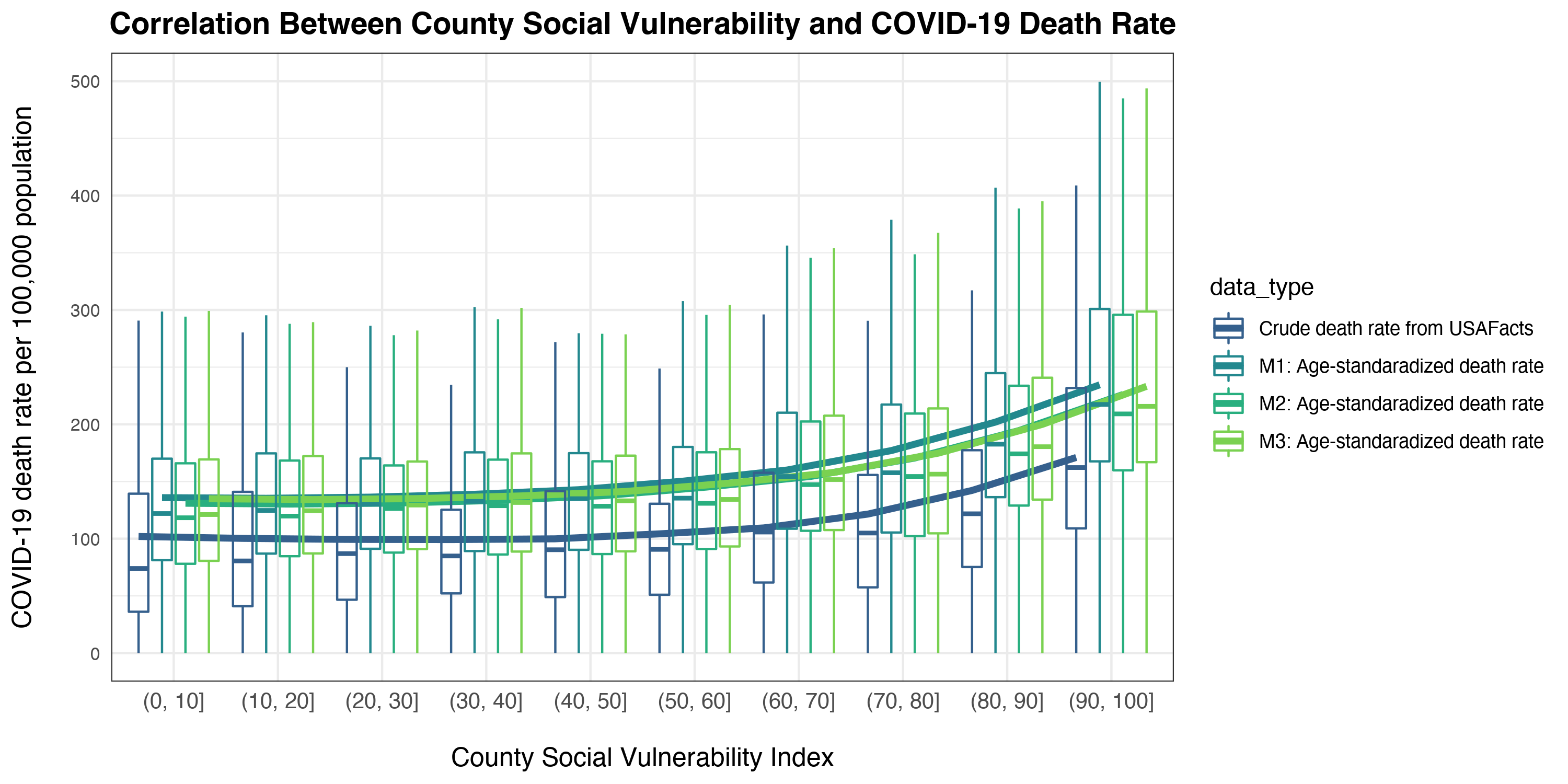

Supplement: S6 Fig — (TIFF) [file pone.0288961.s011.tiff]
